# Elevated GFI1 in Alveolar Macrophages Suppresses ACOD1 Expression and Exacerbates Lipopolysaccharide‐Induced Lung Injury in Obesity

**DOI:** 10.1002/advs.202413546

**Published:** 2025-02-08

**Authors:** Jingyue Ma, Yichan Ao, Zhen Yue, Zhiqiao Wang, Xiangyu Hou, Hongbin Li, Hanbing Wang, Siqing Luo, Jianyu He, Zikun Duan, Ling Liu, Ke Wei

**Affiliations:** ^1^ Department of Anesthesiology The First Affiliated Hospital of Chongqing Medical University Chongqing 400016 China; ^2^ Department of Anesthesiology Xinjiang Uygur Autonomous Region Changji People's Hospital Changji 831100 China

**Keywords:** ACOD1, ARDS, lung injury, macrophages, obesity

## Abstract

To investigate the mechanisms behind the worsening of acute lung injury (ALI) in obesity, transcriptomic sequencing is performed, and significantly reduced mRNA levels of Aconitate Decarboxylase 1 (ACOD1) in the lung tissue of high‐fat diet (HFD) mice are found. Clinical samples are collected, an ALI model is established in HFD mice, and both human and mouse samples are analyzed, revealing a significant decrease in ACOD1 expression in lung tissue and alveolar macrophages in obesity. Further in vivo and in vitro experiments show that ACOD1 knockdown worsens lung injury, inflammation, and oxidative stress, while ACOD1 overexpression alleviates these effects. Moreover, nuclear factor erythroid 2‐related factor 2 (Nrf2) inhibition diminishes the protective effects of ACOD1 overexpression in ALI exacerbated by obesity. Additionally, in the context of obesity, growth factor independent 1 (GFI1) protein levels are elevated in alveolar macrophages, and its knockdown leads to upregulated ACOD1 expression. Therefore, this study suggests that ACOD1 downregulation in alveolar macrophages is a key factor in worsening ALI in obesity, likely driven by GFI1 upregulation.

## Graphical abstract

This study reveals that in alveolar macrophages of high‐fat diet‐induced obese mice, the transcriptional repressor GFI1 is upregulated. This upregulation suppresses ACOD1 expression, which intensifies lipopolysaccharide‐induced lung inflammation and oxidative stress, thereby worsening acute lung injury.

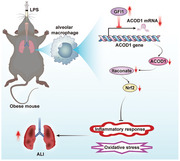

## 1 Introduction

Obesity affects over 890 million people globally, according to the World Health Organization,^[^ ^1^ ^]^ and is a growing public health concern. It is commonly linked to comorbidities such as cardiovascular disease,^[^ ^2^, ^3^ ^]^ diabetes,^[^ ^4^ ^]^ kidney disease,^[^ ^5^ ^]^ cancer,^[^ ^6^, ^7^ ^]^ and various respiratory disorders.^[^ ^8^, ^9^, ^10^, ^11^, ^12^, ^13^, ^14^ ^]^

Acute lung injury (ALI) is triggered by various pathological factors and is characterized by uncontrolled oxidative stress, pulmonary edema, and inflammatory cell infiltration.^[^ ^15^ ^]^ As ALI progresses, it can develop into acute respiratory distress syndrome (ARDS), a leading cause of mortality in critically ill patients. Obesity has been shown to significantly increase the severity of ARDS, prolong hospitalization, and complicate disease management.^[^ ^9^, ^16^ ^]^ The chronic inflammation, lipid metabolism disorders, and abnormal immune responses associated with obesity may collectively impact the lungs, accelerating disease progression and complicating treatment.^[^ ^8^ ^]^ Previous research has demonstrated that obesity worsens lung injury caused by factors such as ozone, PM2.5, ischemia‐reperfusion, lipopolysaccharide (LPS), and sepsis,^[^ ^17^, ^18^, ^19^, ^20^, ^21^, ^22^, ^23^, ^24^, ^25^ ^]^ suggesting distinct pathological mechanisms in ALI in the context of obesity. To identify potential molecular targets, we previously conducted transcriptomic sequencing, which revealed a significant reduction in Aconitate Decarboxylase 1 (ACOD1) mRNA levels in the lung tissue of high‐fat diet (HFD) mice.^[^ ^23^ ^]^

ACOD1, also known as IRG1 (Immunoresponsive Gene 1), was first identified in LPS‐stimulated mouse macrophages.^[^ ^26^ ^]^ As a key immunoregulatory enzyme, ACOD1 has garnered significant attention in recent years. It is primarily expressed in immune cells such as macrophages, with its expression notably upregulated during inflammation and infection.^[^ ^27^ ^]^ ACOD1's main function is to catalyze the conversion of *cis*‐aconitate from the tricarboxylic acid cycle into itaconate.^[^ ^28^ ^]^ Itaconate, a versatile metabolite, has been found to exert potent regulatory effects on inflammation by mechanisms such as inhibiting the IκBζ/ATF3 axis,^[^ ^29^ ^]^ activating nuclear factor erythroid 2‐related factor 2 (Nrf2),^[^ ^30^ ^]^ suppressing inflammasome activation,^[^ ^31^ ^]^ inhibiting aerobic glycolysis,^[^ ^32^ ^]^ and blocking succinate dehydrogenase (SDH),^[^ ^33^, ^34^ ^]^ thereby providing both anti‐inflammatory and antioxidant effects. Studies have shown that ACOD1 plays protective roles in various pulmonary diseases through its anti‐inflammatory and antioxidant functions.^[^ ^35^, ^36^, ^37^, ^38^, ^39^, ^40^ ^]^ However, it remains unclear whether obesity affects ALI progression by regulating ACOD1.

In this study, we identified the downregulation of ACOD1 in alveolar macrophages in the context of obesity, confirmed its protective role in LPS‐induced ALI in HFD mice, and preliminarily elucidated its potential mechanisms of action and regulation.

## 2 Results

### 2.1 Pulmonary ACOD1 Expression Was Decreased in the Context of Obesity

**Figure 1:**
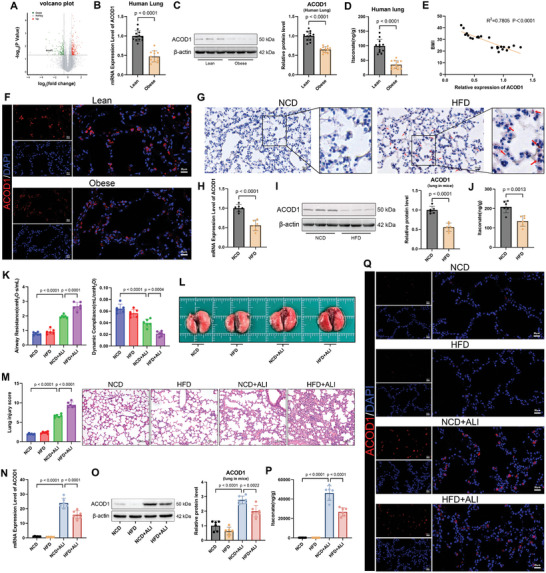
Reduction of ACOD1 in lung tissue in the context of obesity. A) Volcano plot analysis of differential gene expression in the lung tissue of HFD mice. Red dots represent up‐regulated genes, and green dots represent down‐regulated genes. B) ACOD1 mRNA levels in lung tissue from normal‐weight and obese patients (*n* = 12). C) ACOD1 protein levels in lung tissue from normal‐weight and obese patients (*n* = 12). D) Itaconate levels in lung tissue from normal‐weight and obese patients (*n* = 12). E) Correlation of ACOD1 expression with BMI in human lung tissue. F) Immunofluorescence of ACOD1 in lung tissue from normal‐weight and obese patients (*n* = 3). Scale bar: 20 µm. G) Oil red O staining of lung tissue from NCD and HFD mice(*n* = 3). Red arrows indicate typical lipid droplets. Scale bar: 20 µm. H) ACOD1 mRNA levels in lung tissue from NCD and HFD mice (*n* = 6). I) ACOD1 protein levels in lung tissue from NCD and HFD mice (*n *= 6). J) Itaconate levels in lung tissue from NCD and HFD mice (*n* = 6). K) Airway resistance and lung compliance in NCD and HFD mice 24 h after LPS (*n* = 6). L) Lung images of mice with LPS‐induced lung injury. M) Representative H&E‐stained lung tissue sections of LPS‐induced ALI and lung injury scoring (*n* = 6). Scale bar: 50 µm. N) RT‐qPCR analysis of ACOD1 mRNA levels in lung tissue of mice with LPS‐induced ALI (*n* = 6). O) Western blot analysis of ACOD1 protein levels in lung tissue of mice with LPS‐induced ALI. (*n* = 6). P) Itaconate levels in lung tissue of mice with LPS‐induced ALI (*n* = 6). Q) Immunofluorescence staining of ACOD1 in lung tissue sections of mice(*n* = 3). Scale bar: 20 µm. Data are expressed as mean ± SD.

In our previous study, transcriptomic sequencing of lung tissue from HFD mice revealed significantly lower ACOD1 mRNA levels compared to normal mice (**Figure** **1**A).^[^ ^23^ ^]^ To validate this finding in humans, we collected lung tissue samples from both normal‐weight and patients with obesity. ACOD1 mRNA levels were significantly reduced in the lung tissue of patients with obesity compared to normal‐weight patients (Figure 1B). Western blot and immunofluorescence analyses further confirmed that ACOD1 protein levels were markedly lower in the lung tissue of patients with obesity (Figure 1C,F). Similarly, the ACOD1 metabolite, itaconate, was also significantly reduced in the lung tissue of patients with obesity (Figure 1D). Additionally, we observed a negative correlation between ACOD1 expression and BMI, blood lipid, and blood glucose levels (Figure 1E; Figure S1A–C, Supporting Information).

To further confirm the effects of obesity on ACOD1 expression, we established a mouse model of obesity by feeding mice an HFD for 16 weeks. Compared to the normal chow diet (NCD) group, HFD mice exhibited significantly increased body weight (Figure S1D,E, Supporting Information), elevated lipid levels (Figure S1F, Supporting Information), and higher fasting blood glucose (Figure S1G, Supporting Information). Impaired glucose tolerance was also observed in HFD mice, as indicated by Intraperitoneal Glucose Tolerance Test (IPGTT) results (Figure S1H, Supporting Information). Oil Red O staining demonstrated more pronounced lipid accumulation in the heart, lung, liver, and kidney of HFD mice compared to NCD mice (Figure 1G; Figure S1I, Supporting Information). Consistent with the findings in human samples, ACOD1 mRNA and protein levels, as well as itaconate concentrations, were significantly reduced in the lung tissue of HFD mice (Figure 1H–J).

Previous studies have shown that ACOD1 is notably upregulated during inflammation.^[^ ^27^ ^]^ To explore the role of ACOD1 in LPS‐induced ALI in the context of obesity, we established an ALI model in mice via intratracheal LPS administration. Using the Buxco Pulmonary Function Testing system to assess lung function (Figure S1J, Supporting Information), the results indicated that LPS exposure led to a significant increase in airway resistance and a reduction in lung compliance, with HFD mice exhibiting more severe pulmonary dysfunction compared to NCD mice (Figure 1K). Gross examination of lung tissue further revealed obvious congestion and edema following LPS exposure, with more severe damage in HFD mice (Figure 1L). Pathological analysis using H&E staining corroborated these findings, showing that LPS exposure caused substantial pathological damage, including alveolar structural destruction, thickened alveolar walls, and increased inflammatory cell infiltration. These pathological alterations were more severe in HFD mice than in NCD mice (Figure 1M). Correspondingly, LPS exposure resulted in increased protein concentration in bronchoalveolar lavage fluid (BALF), a higher lung wet/dry (W/D) ratio, and elevated lactate dehydrogenase (LDH) activity (Figure S1K, Supporting Information), with these effects being markedly more pronounced in HFD mice. Inflammatory cytokine levels in BALF and serum further confirmed the exacerbation of inflammation induced by LPS exposure. Specifically, LPS exposure significantly elevated the levels of pro‐inflammatory cytokines IL‐1β, IL‐6, and TNF‐α, as well as the anti‐inflammatory cytokine IL‐10. However, compared to NCD mice, HFD mice exhibited more pronounced increases in pro‐inflammatory cytokines and a relatively smaller increase in IL‐10, indicating a heightened inflammatory response and impaired anti‐inflammatory regulation in HFD mice. The changes in serum cytokines mirrored those in BALF, highlighting the consistency between systemic and pulmonary inflammatory responses (Figure S1L,M, Supporting Information). Oxidative stress markers in lung tissue provided further evidence supporting these findings. LPS exposure significantly increased the levels of reactive oxygen species (ROS) and malondialdehyde (MDA) in both NCD and HFD groups, with the highest levels observed in the HFD +ALI group. Conversely, antioxidant markers, including superoxide dismutase (SOD), catalase (CAT), and glutathione (GSH), were significantly reduced in both LPS‐exposed groups, with more pronounced reductions in the HFD +ALI group (Figure S1N, Supporting Information). In conclusion, LPS exposure induced significant pulmonary dysfunction, tissue damage, inflammation, and oxidative stress, with these effects being more severe in HFD mice compared to NCD mice, consistent with our previous studies highlighting the heightened susceptibility of HFD mice to LPS‐induced lung injury.^[^ ^22^, ^24^ ^]^ ACOD1 mRNA and protein levels, along with its downstream metabolite itaconate, were significantly upregulated in both groups following LPS exposure. However, the increase in HFD mice remained consistently lower than in NCD mice (Figure 1N–P). This pattern was further confirmed by immunofluorescence analysis (Figure 1Q). Additionally, previous studies have shown lower ACOD1 expression in the liver of obese individuals compared to those of normal weight.^[^ ^41^ ^]^ Consistent with these findings, our study also revealed varying degrees of reduced ACOD1 levels in the heart, liver, and kidneys of HFD mice (Figure S1O, Supporting Information). While these results suggest obesity‐related reductions in ACOD1 expression across multiple organs, this study specifically focuses on the downregulation of ACOD1 in the lung and its contribution to the exacerbation of ALI following LPS exposure in the context of obesity.

### 2.2 Pulmonary ACOD1 as a Critical Modulator of LPS‐Induced Lung Injury, Oxidative Stress, and Inflammation

#### 2.2.1 Pulmonary ACOD1 Knockdown Exacerbated LPS‐Induced Lung Injury, Oxidative Stress, and Inflammation

**Figure 2:**
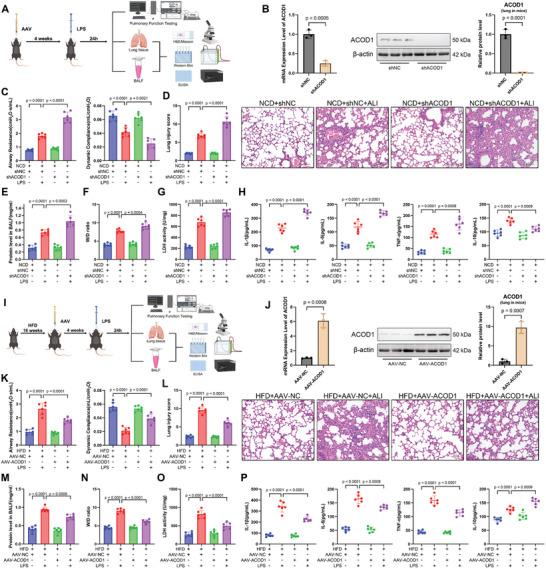
Regulation of Lung ACOD1 Modulates LPS‐Induced Lung Injury. A) Schematic representation of the experimental procedure in NCD mice. AAV‐shACOD1 (5 × 10¹¹ vg per mouse) was administered via airway, followed by LPS (100 µg per mouse) after 4 weeks to induce ALI. Assessments were performed 24 h post‐LPS for lung function, injury severity, inflammation, and oxidative stress. B) mRNA and protein expression levels of ACOD1 in mouse lung tissue 4 weeks after infection with adeno‐associated virus shACOD1 (*n* = 3). C) Airway resistance and lung compliance in NCD mice with ACOD1 knockdown during LPS‐induced lung injury, measured using the Buxco Pulmonary Function Test System (*n* = 6). D) H&E staining and lung injury scores of lung tissue in NCD mice (*n* = 6). Scale bar: 50 µm. E) Protein concentration in BALF of NCD mice across groups (*n* = 6). F) Lung tissue dry‐to‐wet ratio in each group of NCD mice (*n* = 6). G) LDH activity in lung tissue of NCD mice across groups (*n* = 6). H) Protein levels of IL‐1β, IL‐6, TNF‐α, and IL‐10 in BALF of NCD mice were measured by ELISA (*n* = 6). I) Schematic representation of the experimental procedure in HFD mice. Mice were fed a high‐fat diet for 16 weeks to induce obesity, followed by airway administration of AAV‐ACOD1 (5 × 10¹¹ vg per mouse). After 4 weeks, LPS (100 µg per mouse) was administered to induce ALI. Assessments were performed 24 h post‐LPS for lung function, injury severity, inflammation, and oxidative stress. J) mRNA and protein expression levels of ACOD1 in mouse lung tissue 4 weeks after infection with adeno‐associated virus AAV‐ACOD1 (*n* = 3). K) Airway resistance and lung compliance in HFD mice with ACOD1 overexpression during LPS‐induced lung injury, measured using the Buxco Pulmonary Function Test System (*n* = 6). L) H&E staining and lung injury scores of lung tissue in HFD mice (*n* = 6). Scale bar: 50 µm. M) Protein concentration in BALF of HFD mice (*n* = 6). N) Lung tissue dry‐to‐wet ratio of HFD mice (*n *= 6). N) LDH activity in lung tissue of HFD mice (*n* = 6). O) Protein levels of IL‐1β, IL‐6, TNF‐α, and IL‐10 in BALF of HFD mice were measured by ELISA (*n* = 6). Data are expressed as mean ± SD.

To specifically focus on the role of ACOD1 in the lungs rather than system‐wide effects, we selectively knocked down ACOD1 expression in the lungs of normal‐weight mice via airway administration of adeno‐associated virus (sh‐ACOD1) (5 × 10¹^2^ vg ml^−1^, 100 µL per mouse, equivalent to 5 × 10¹¹ vg per mouse) (**Figure** **2**A). Four weeks post‐infection, ACOD1 mRNA and protein levels were significantly reduced in the lungs (Figure 2B). After LPS exposure, typical features of ALI were observed. Pulmonary function tests demonstrated that ACOD1 knockdown mice exhibited more severe lung function impairment, as evidenced by increased airway resistance and decreased lung compliance compared to control mice (Figure 2C). H&E staining showed significantly exacerbated tissue damage in ACOD1 knockdown mice following LPS exposure compared to controls, characterized by greater structural destruction, thickened alveolar walls, and increased inflammatory cell infiltration (Figure 2D). Furthermore, ACOD1 knockdown mice displayed markedly elevated total protein content in BALF, W/D ratio, and LDH activity compared to controls, corroborating the heightened severity of lung injury in ACOD1 knockdown mice following LPS exposure (Figure 2E–G).

Further analysis of inflammatory cytokines in BALF, serum, and oxidative stress markers in lung tissue indicated that pulmonary ACOD1 knockdown significantly exacerbated LPS‐induced inflammation and oxidative stress. Pro‐inflammatory cytokines IL‐1β, IL‐6, and TNF‐α were significantly elevated in both BALF and serum, while the anti‐inflammatory cytokine IL‐10 was reduced (Figure 2H; Figure S2A, Supporting Information). Oxidative stress analysis of lung tissue revealed increased levels of ROS and MDA, accompanied by reduced activities of antioxidant enzymes, including SOD, CAT, and GSH (Figure S2B, Supporting Information), further highlighting the heightened oxidative stress in pulmonary ACOD1 knockdown mice. These results suggest that ACOD1 plays a critical role in mitigating inflammation and oxidative stress following LPS exposure. In summary, airway administration of sh‐ACOD1 specifically knocked down ACOD1 expression in the lungs, which exacerbated LPS‐induced lung function decline and tissue damage by enhancing inflammation and oxidative stress. These findings suggest that lung‐specific ACOD1 plays a critical role in modulating LPS‐induced ALI, and its downregulation through airway targeting leads to increased inflammation, oxidative stress, and more severe lung injury.

#### 2.2.2 Overexpression of Pulmonary ACOD1 Alleviated LPS‐Induced Lung Injury, Oxidative Stress, and Inflammation in HFD Mice

First, we specifically overexpressed ACOD1 in the lungs of HFD mice, fed a high‐fat diet for 16 weeks, via airway administration of adeno‐associated virus (AAV‐ACOD1, 5 × 10¹^2^ vg ml^−1^; 100 µL mouse^−1^, 5 × 10¹¹ vg mouse^−1^) to counteract the obesity‐induced reduction in ACOD1 levels (Figure 2I), with a particular focus on its effects within the lungs. Four weeks after infection, pulmonary ACOD1 expression in HFD mice was significantly elevated (Figure 2J). In the LPS‐induced ALI model, this lung‐specific overexpression of ACOD1 demonstrated significant protective effects. Pulmonary function tests demonstrated that ACOD1 overexpression alleviated LPS‐induced lung dysfunction in HFD mice, as evidenced by reduced airway resistance and improved lung compliance compared to HFD mice exposed to LPS without ACOD1 overexpression (Figure 2K). Consistent with these observations, H&E staining showed attenuated tissue damage, characterized by reduced structural destruction and diminished inflammatory cell infiltration (Figure 2L). Furthermore, ACOD1‐overexpressing HFD mice displayed lower total protein content in BALF, W/D ratio, and LDH activity compared to controls, confirming the mitigated severity of lung injury following LPS exposure (Figure 2M–O).

Further analysis of inflammatory cytokines in BALF and serum, as well as oxidative stress markers in lung tissue, demonstrated that pulmonary ACOD1 overexpression significantly alleviated LPS‐induced inflammation and oxidative stress in HFD mice. Specifically, ACOD1 overexpression in the lungs of HFD mice reduced LPS‐induced elevations in pro‐inflammatory cytokines IL‐1β, IL‐6, and TNF‐α in both BALF and serum, while significantly increasing the anti‐inflammatory cytokine IL‐10 (Figure 2P; Figure S2C, Supporting Information). In lung tissue, oxidative stress analysis revealed decreased levels of ROS and MDA, accompanied by increased activities of antioxidant enzymes, including SOD, CAT, and GSH (Figure S2D, Supporting Information), highlighting the role of ACOD1 overexpression in mitigating oxidative stress and inflammation caused by LPS exposure.

It is well established that ACOD1 exerts its anti‐inflammatory and antioxidant effects primarily through its downstream product, itaconate. To further explore this mechanism, we used 4‐OI, a cell‐permeable itaconate derivative widely applied in studies of itaconate's anti‐inflammatory and antioxidant properties.^[^ ^42^ ^]^ Following the manufacturer's instructions and previous studies,^[^ ^43^ ^]^ 4‐OI was dissolved in 10% DMSO + 40% PEG400 + 5% Tween‐80 + 45% saline. HFD mice were administered 4‐OI (50 mg k^−1^g) via intraperitoneal injection 24 and 2 h prior to LPS exposure, and assessments were made 24 h after LPS treatment. In line with the effects of ACOD1 overexpression, exogenous itaconate supplementation partially improved lung function (Figure S4A, Supporting Information), reduced lung injury (Figure S4B–E, Supporting Information), and decreased inflammation (Figure S4F,G, Supporting Information) and oxidative stress (Figure S4H, Supporting Information) in HFD mice following LPS exposure.

In conclusion, airway‐targeted overexpression of ACOD1 and its downstream metabolite itaconate confer significant protective effects against the heightened severity of LPS‐induced lung injury observed in HFD mice, highlighting their potential as a basis for future pulmonary‐specific therapeutic investigations.

### 2.3 ACOD1 Overexpression‐Mediated Protection in Obesity‐Exacerbated ALI Was Macrophage‐Dependent

**Figure 3:**
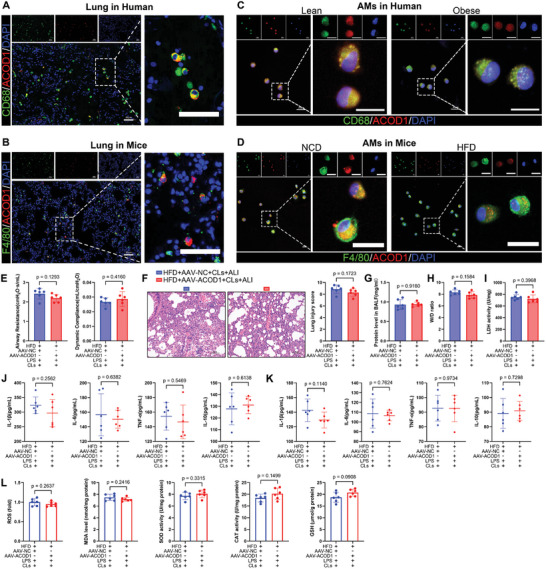
Alveolar macrophages play a major role in ACOD1‐mediated protection against ALI in obesity. A) Dual‐labeled immunofluorescence showing colocalization of ACOD1 (red) and CD68 (green) in human lung tissue (*n* = 3). Scale bar: 50 µm. B) Dual‐labeled immunofluorescence showing colocalization of ACOD1 (red) and F4/80 (green) in mouse lung tissue (*n* = 3). Scale bar: 50 µm. C) Dual‐labeled immunofluorescence showing colocalization of ACOD1 (red) and CD68 (green) in primary alveolar macrophages from normal‐weight patients and patients with obesity (*n* = 3). Scale bar: 20 µm. D) Dual‐labeled immunofluorescence showing colocalization of ACOD1(red) and F4/80 (green) in primary alveolar macrophages from NCD and HFD mice(*n* = 3). Scale bar: 20 µm. E) Airway resistance and lung compliance in different groups after macrophage depletion, measured using the Buxco Pulmonary Function Test System (*n* = 6). F) H&E staining and lung injury score in lung tissue from each group (*n* = 6). Scale bar: 50 µm. G) Protein concentration in BALF from each group (*n* = 6). H) Lung tissue dry‐to‐wet ratio in each group (*n *= 6). I) LDH activity in lung tissue from each group (*n* = 6). J) Levels of IL‐1β, IL‐6, TNF‐α, and IL‐10 in BALF were measured by ELISA (*n* = 6). K) Levels of IL‐1β, IL‐6, TNF‐α, and IL‐10 in serum were measured by ELISA (*n* = 6). L) Levels of ROS, MDA, SOD, CAT, and GSH in lung tissue were measured using commercial kits (*n* = 6). Data are expressed as mean ± SD.

According to previous studies, ACOD1 is primarily expressed in immune cells, particularly macrophages.^[^ ^27^ ^]^ To determine its cellular localization and role in our model, we performed dual immunofluorescence staining on human and mouse lung tissues to assess ACOD1 colocalization with macrophage markers (CD68+ in human tissue, F4/80+ in mouse tissue). Results revealed that ACOD1 is predominantly expressed in lung macrophages (**Figure** **3**A,B). Immunofluorescence staining of primary alveolar macrophages, isolated from bronchoalveolar lavage fluid, further confirmed ACOD1 expression in alveolar macrophages, with lower levels observed in obese individuals compared to normal controls (Figure 3C,D)

Although alveolar macrophages are known contributors to acute lung injury,^[^ ^44^, ^45^, ^46^ ^]^ their role in ACOD1‐mediated protection against the exacerbated ALI observed in HFD mice remains unclear. To investigate this, we employed intratracheal clodronate liposomes (CLs) to deplete alveolar macrophages in mice. Immunofluorescence confirmed that a single dose of CLs (100 µL per mouse) administered via airway effectively depleted lung macrophages,^[^ ^47^ ^]^ whereas control liposomes (CL‐PBS) showed no significant effect (Figure S3A, Supporting Information). In our previous study, we demonstrated that ACOD1 overexpression in the lungs of HFD mice, achieved via AAV‐ACOD1, mitigated obesity‐exacerbated lung injury. Therefore, we administered CLs to both AAV‐ACOD1 and AAV‐NC groups of HFD mice, followed by LPS exposure to induce acute lung injury. Consequently, the anti‐inflammatory, antioxidative, and tissue‐protective effects of ACOD1 were largely diminished in HFD mice with alveolar macrophage depletion. Specifically, no differences were observed between the two groups in lung dysfunction (Figure 3E), tissue damage (Figure 3F), BALF protein concentration (Figure 3G), W/D ratio (Figure 3H), LDH activity (Figure 3I), cytokine levels in BALF and serum (Figure 3J,K), or oxidative stress (Figure 3L).

Given the role of neutrophils in ALI,^[^ ^48^, ^49^ ^]^ We used intratracheal instillation of anti‐Ly6G antibodies (100 µg, per mouse) to deplete pulmonary neutrophils.^[^ ^50^ ^]^ Immunofluorescence confirmed that anti‐Ly6G antibodies, but not the isotype control (rat IgG2a), effectively depleted pulmonary neutrophils (Figure S3B, Supporting Information). Building on this, we treated the AAV‐NC and AAV‐ACOD1 groups of HFD mice with anti‐Ly6G antibody to deplete pulmonary neutrophils, followed by intratracheal LPS administration to induce lung injury. Analysis revealed that ACOD1 overexpression continued to exert protective effects after neutrophil depletion (Figure S3C–J, Supporting Information).

In conclusion, these results suggest that the alveolar macrophages appeared to be the major effectors responsible for ACOD1‐mediated protection against exacerbated ALI in HFD mice.

### 2.4 ACOD1 Deficiency Aggravated LPS‐Induced Macrophage Inflammation and Oxidative Stress

**Figure 4:**
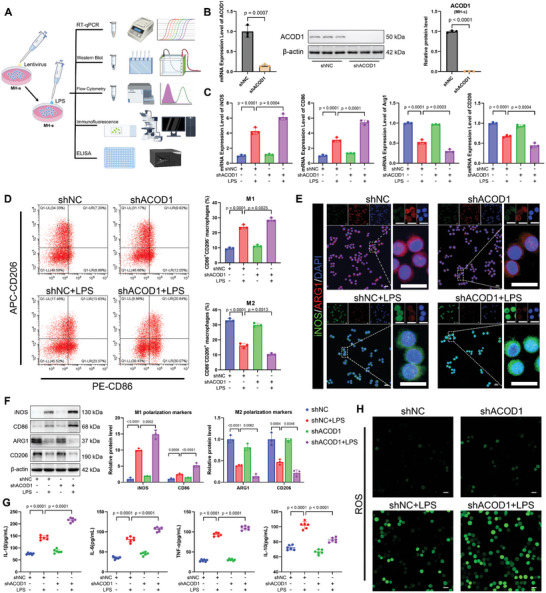
Knockdown of ACOD1 increases LPS‐induced inflammation and oxidative stress in MH‐s macrophages. A) Schematic representation of the experimental procedure in MH‐s cells. MH‐s cells were stably transduced with lentiviral shACOD1 to knock down ACOD1 expression. After 24‐hour treatment with LPS (100 ng mL^−1^), inflammation and oxidative stress levels were assessed. B) mRNA and protein expression levels of ACOD1 in MH‐s cells after shACOD1 transfection (*n* = 3). C) mRNA levels of M1 macrophage markers (iNOS, CD86) and M2 macrophage markers (Arg‐1, CD206) in MH‐s cells detected by RT‐qPCR (*n* = 3). D) Flow cytometry analysis of the proportion of M1 macrophages (CD86+CD206−) and M2 macrophages (CD86−CD206+) in MH‐s cells (*n* = 3). E) Immunofluorescence detection of protein levels of M1 macrophage marker (iNOS, green) and M2 macrophage marker (Arg‐1, red) in MH‐s cells (*n* = 3). Scale bar: 20 µm. F) Western blot analysis of iNOS, CD86, Arg1, and CD206 in MH‐s cells (*n* = 3). G) levels of IL‐1β, IL‐6, TNF‐α, and IL‐10 in the supernatant of cell culture medium were measured by ELISA (*n* = 6). H) Immunofluorescent labeling of ROS in MH‐s cells (*n* = 3). Scale bar: 20 µm. Data are expressed as mean ± SD.

To further investigate the role of ACOD1 in LPS‐induced inflammation and oxidative stress in alveolar macrophages, we generated a stable ACOD1‐knockdown MH‐s cell line using lentiviral shACOD1 (MOI = 50) (**Figure** **4**A). ACOD1 mRNA and protein levels were significantly reduced in these knockdown cells (Figure 4B). Upon LPS exposure, mRNA levels of M1 macrophage markers iNOS and CD86 were significantly upregulated, accompanied by a notable downregulation of mRNA levels of M2 macrophage markers Arg‐1 and CD206. This M1/M2 polarization imbalance was further aggravated in ACOD1‐knockdown cells, with even higher expression of M1 markers and further suppression of M2 markers (Figure 4C). Flow cytometry was employed to quantify the proportions of M1 macrophages (CD86⁺CD206⁻) and M2 macrophages (CD86⁻CD206⁺) (Figure 4D). Immunofluorescence staining was utilized to detect the protein expression of the M1 marker iNOS and the M2 marker Arg‐1 (Figure 4E). Additionally, Western blot analysis was conducted to measure the expression levels of iNOS, Arg‐1, CD86, and CD206 proteins (Figure 4F). Combined, these analyses demonstrated that LPS exposure upregulated the expression of M1 polarization markers (iNOS and CD86) while downregulating M2 polarization markers (Arg‐1 and CD206) in macrophages at the protein level. Notably, ACOD1 knockdown further exacerbated this LPS‐induced polarization shift.

LPS exposure significantly increased pro‐inflammatory cytokines (IL‐6, IL‐1β, TNF‐α) and the anti‐inflammatory cytokine IL‐10 in macrophages, along with elevated ROS and MDA levels and decreased antioxidant enzyme activities (SOD, CAT, GSH). In ACOD1‐knockdown cells, pro‐inflammatory cytokines (IL‐6, IL‐1β, TNF‐α) were further elevated, while IL‐10 levels were lower compared to control cells exposed to LPS, reflecting an intensified pro‐inflammatory response and a weakened anti‐inflammatory response. Oxidative stress was also aggravated, with higher ROS and MDA levels and further reductions in antioxidant enzyme activities (SOD, CAT, GSH) (Figure 4G,H; Figure S5A, Supporting Information).

In conclusion, these findings suggest that ACOD1 deficiency intensifies LPS‐induced inflammation and oxidative stress in macrophages.

### 2.5 ACOD1 Overexpression in a High‐Fat Environment Alleviated LPS‐Induced Macrophage Inflammation and Oxidative Stress

To simulate a high‐fat environment in vitro, MH‐s cells were co‐cultured with adipocytes differentiated from 3T3‐L1 preadipocytes, leading to a significant reduction in ACOD1 expression in MH‐s cells (Figure S5B, Supporting Information). Upon LPS exposure, ACOD1 levels in MH‐s cells markedly increased (Figure S5C, Supporting Information); however, they remained lower in the co‐culture group compared to the control group (Figure S5D, Supporting Information)

**Figure 5:**
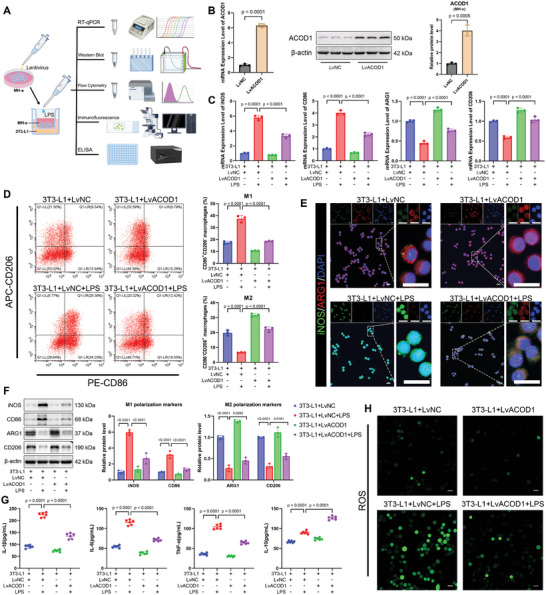
Overexpression of ACOD1 alleviates LPS‐induced inflammation and oxidative stress in MH‐s macrophages in a high‐lipid environment. A) Schematic representation of the experimental procedure in MH‐s cells. MH‐s cells were transduced with LvACOD1 to establish stable ACOD1 overexpression cell lines. These cells were co‐cultured with adipocytes for 48 h, followed by LPS treatment (100 ng mL^−1^) for 24 h to evaluate inflammation and oxidative stress levels. B) mRNA and protein expression levels of ACOD1 in MH‐s cells after LvACOD1 transfection (*n* = 3). C) RT‐qPCR detection of M1 markers (iNOS, CD86) and M2 markers (Arg‐1, CD206) in MH‐s cells under a high‐lipid environment (*n* = 3). D)Flow cytometry analysis of the proportion of M1 macrophages (CD86+CD206−) and M2 macrophages (CD86−CD206+) in MH‐s cells under a high‐lipid environment (*n* = 3). E) Immunofluorescence detection of protein levels of M1 macrophage marker (iNOS, green) and M2 macrophage marker (Arg‐1, red) in MH‐s cells under a high‐lipid environment (*n *= 3). Scale bar: 20 µm. F) Western blot analysis of iNOS, CD86, Arg1, and CD206 in MH‐s cells under a high‐lipid environment (*n* = 3). G) levels of IL‐1β, IL‐6, TNF‐α, and IL‐10 in the supernatant of cell culture medium were measured by ELISA (*n* = 6). H) Immunofluorescent labeling of ROS in MH‐s cells under a high‐lipid environment (*n* = 3). Scale bar: 20 µm. Data are expressed as mean ± SD.

Subsequently, we generated a stable ACOD1‐overexpressing MH‐s cell line using lentivirus (Lv‐ACOD1, MOI = 50) (**Figure** **5**A) and confirmed a significant increase in ACOD1 mRNA and protein levels (Figure 5B). Both ACOD1‐overexpressing cells and control cells were co‐cultured with adipocytes to simulate a high‐fat environment, followed by LPS exposure under identical conditions. In this high‐fat environment, LPS significantly upregulated M1 macrophage markers (iNOS, CD86) and downregulated M2 markers (Arg‐1, CD206), indicating that macrophages in a high‐fat environment are susceptible to LPS‐induced pro‐inflammatory polarization. However, in ACOD1‐overexpressing cells co‐cultured with adipocytes, this polarization shift was markedly mitigated, as evidenced by the reversal of M1 marker upregulation and M2 marker downregulation at the mRNA level (Figure 5C). These results were further corroborated by flow cytometry, immunofluorescence, and Western blot analysis, which collectively confirmed the observed polarization changes (Figure 5D–F).

In the high‐fat environment, LPS exposure significantly exacerbated inflammation and oxidative stress in MH‐s cells, as evidenced by elevated levels of pro‐inflammatory cytokines (IL‐1β, IL‐6, TNF‐α), the anti‐inflammatory cytokines (IL‐10), and oxidative stress markers (ROS, MDA), alongside decreased activities of antioxidant enzymes (SOD, CAT, GSH). Notably, ACOD1 overexpression in MH‐s cells notably mitigated these LPS‐induced inflammatory and oxidative responses under high‐fat conditions (Figure 5G,H; Figure S5E, Supporting Information).

Furthermore, we observed that the addition of 62.5µm 4‐OI to the co‐culture of MH‐s cells and adipocytes, as reported in previous studies,^[^ ^43^ ^]^ followed by LPS exposure, resulted in a reduction of M1 macrophage proportions, an increase in M2 macrophage proportions (Figure S4I–L, Supporting Information), and a significant attenuation of LPS‐induced inflammation and oxidative stress (Figure S4M–O, Supporting Information).

These findings suggest that in an in vitro high‐fat environment, ACOD1 expression is reduced in alveolar macrophages. However, both ACOD1 overexpression and exogenous itaconate supplementation effectively alleviate LPS‐induced macrophage inflammation and oxidative stress.

### 2.6 In Vivo Regulation of ACOD1 Expression in Lung Macrophages

We specifically knocked down ACOD1 expression in lung macrophages of normal‐weight mice by intratracheal administration of adeno‐associated virus with an F4/80‐specific promoter (AAV‐F4/80‐shACOD1, 5 × 10¹^2^ vg ml^−1^; 100 µl mouse^−1^, equivalent to 5 × 10¹¹ vg mouse^−1^). Four weeks after AAV infection, double immunofluorescence staining for macrophages (F4/80) and ACOD1 in lung tissues showed that ACOD1 expression in lung macrophages was significantly reduced in the knockdown group compared to the control group (Figure S6A, Supporting Information). Furthermore, primary alveolar macrophages extracted from BALF were analyzed by qPCR and immunofluorescence staining, which confirmed significantly decreased ACOD1 expression in alveolar macrophages in the knockdown group compared to the control group (Figure S6B,C, Supporting Information).

**Figure 6:**
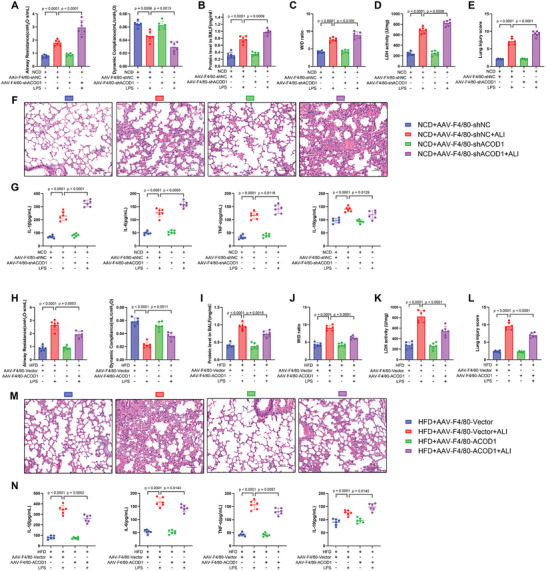
Targeted regulation of macrophage ACOD1 modulates LPS‐induced lung injury. A) Airway resistance and lung compliance in NCD mice with macrophage‐targeted ACOD1 knockdown during LPS‐induced lung injury (*n *= 6). B) Protein concentration in BALF of NCD mice with macrophage‐targeted ACOD1 knockdown (*n *= 6). C) Lung tissue dry‐to‐wet ratio of NCD mice with macrophage‐targeted ACOD1 knockdown (*n* = 6). D) LDH activity in lung tissue of NCD mice with macrophage‐targeted ACOD1 knockdown (*n* = 6). E,F) H&E staining and lung injury scores of lung tissue in NCD mice with macrophage‐targeted ACOD1 knockdown (*n* = 6). Scale bar: 50 µm. G) Levels of IL‐1β, IL‐6, TNF‐α, and IL‐10 in BALF of NCD mice with macrophage‐targeted ACOD1 knockdown (*n* = 6). H) Airway resistance and lung compliance in HFD mice with macrophage‐targeted ACOD1 overexpression during LPS‐induced lung injury (*n* = 6). I) Protein concentration in BALF of HFD mice with macrophage‐targeted ACOD1 overexpression (*n* = 6). J) Lung tissue dry‐to‐wet ratio of HFD mice with macrophage‐targeted ACOD1 overexpression (*n* = 6). K) LDH activity in lung tissue of HFD mice with macrophage‐targeted ACOD1 overexpression (*n* = 6). L,M) H&E staining and lung injury scores of lung tissue in HFD mice with macrophage‐targeted ACOD1 overexpression (*n* = 6). Scale bar: 50 µm. N) Levels of IL‐1β, IL‐6, TNF‐α, and IL‐10 in BALF of HFD mice with macrophage‐targeted ACOD1 overexpression (*n *= 6). Data are expressed as mean ± SD.

Following LPS exposure, typical features of acute lung injury (ALI) were observed. Compared with the control group, mice with ACOD1 knockdown exhibited more severe lung function deterioration after LPS exposure, including increased airway resistance and decreased lung compliance (**Figure** **6**A). Moreover, the degree of lung tissue damage was significantly aggravated (Figure 6E,F), accompanied by elevated total protein levels in BALF, increased lung wet‐to‐dry weight ratio (W/D), and elevated lactate dehydrogenase (LDH) levels (Figure 6B–D). Additionally, the analysis of inflammatory cytokines in BALF and serum, as well as oxidative stress markers in lung tissues, further demonstrated that LPS exposure significantly exacerbated inflammation and oxidative stress in mice with ACOD1 knockdown in lung macrophages. Specifically, levels of pro‐inflammatory cytokines IL‐1β, IL‐6, and TNF‐α were significantly increased, while the level of the anti‐inflammatory cytokine IL‐10 was significantly decreased (Figure 6G; Figure S6D, Supporting Information). Simultaneously, ROS and MDA levels in lung tissues were elevated, while the activities of antioxidant enzymes SOD, CAT, and GSH were significantly reduced (Figure S6E, Supporting Information).

Next, we overexpressed ACOD1 in lung macrophages of HFD mice by intratracheal administration of adeno‐associated virus with an F4/80‐specific promoter (AAV‐F4/80‐ACOD1, 5 × 10¹^2^ vg ml^−1^; 100 µl mouse^−1^, equivalent to 5 × 10¹¹ vg mouse^−1^). Four weeks after AAV infection, double immunofluorescence staining for macrophages (F4/80) and ACOD1 in lung tissues showed significantly increased ACOD1 expression in lung macrophages in the overexpression group compared to the control group (Figure S6F, Supporting Information). Additionally, primary alveolar macrophages extracted from BALF were analyzed by q‐PCR and immunofluorescence staining, further confirming significantly elevated ACOD1 expression in alveolar macrophages in the overexpression group compared to the control group (Figure S6G,H, Supporting Information).

Subsequently, these mice were subjected to LPS exposure via intratracheal administration. The results showed that ACOD1 overexpression mitigated lung function deterioration after LPS exposure in the overexpression group compared to the control group (Figure 6H). Moreover, lung injury severity (Figure 6I–M), inflammatory response (Figure 6N; FigureS6I, Supporting Information), and oxidative stress levels (Figure S6J, Supporting Information) were significantly improved in the overexpression group compared to the control group.

In conclusion, by specifically regulating ACOD1 expression in lung macrophages in vivo, we demonstrated that ACOD1 in macrophages plays a critical protective role in obesity‐aggravated lung injury.

### 2.7 Nrf2 Was Essential for the Protective Role of ACOD1 in Exacerbated ALI in the Context of Obesity

Nrf2 has been widely recognized as a downstream target of ACOD1, playing a crucial role in mediating anti‐inflammatory and antioxidant responses.^[^ ^30^, ^41^, ^51^, ^52^, ^53^, ^54^ ^]^ To elucidate the downstream mechanisms of ACOD1 in the context of aggravated lung injury in obesity, we assessed Nrf2 expression levels in both NCD and HFD mice. Baseline Nrf2 expression in the lung tissue of HFD mice was lower than that in normal mice. Following LPS exposure, Nrf2 levels increased significantly in both groups, though the increase was still lower in HFD mice compared to controls (Figure S7A, Supporting Information), reflecting the same trend observed for ACOD1. We next investigated the relationship between ACOD1 and Nrf2 by targeting the regulation of ACOD1 expression specifically in pulmonary macrophages. In lung tissues from mice with macrophage‐ targeted ACOD1 knockdown or overexpression, Nrf2 mRNA and protein levels were significantly reduced in ACOD1‐knockdown NCD mice, while they were markedly elevated in ACOD1‐overexpressing HFD mice (Figure S7B,C, Supporting Information). Similar trends were observed in vitro in MH‐s, where Nrf2 expression correspondingly decreased or increased with ACOD1 knockdown or overexpression (Figure S7E–G, Supporting Information). These findings suggest that ACOD1 in macrophages positively regulates Nrf2 expression and that the more severe lung injury observed in obesity may be linked to macrophage‐specific ACOD1 downregulation, leading to decreased Nrf2 levels and compromised anti‐inflammatory and antioxidant responses.

**Figure 7:**
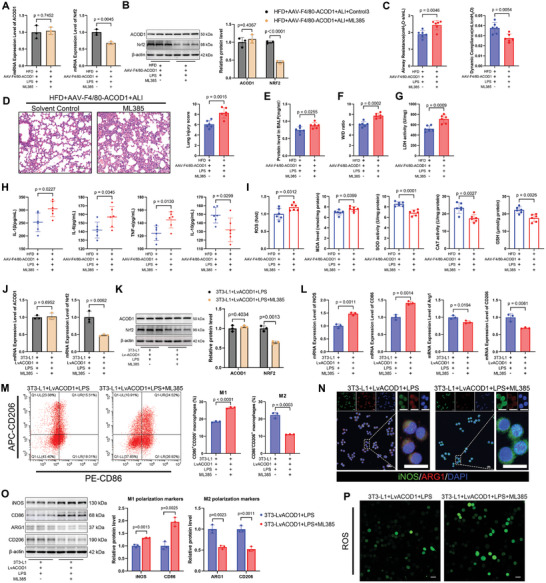
Nrf2 plays a key role in ACOD1‐mediated ALI protection in obesity. A) RT‐qPCR and B) Western blot analysis of ACOD1 and Nrf2 expression in HFD mice with lung macrophage‐specific ACOD1 overexpression treated with ML385 and LPS‐induced lung injury (*n* = 3). C) Airway resistance and lung compliance measured across groups using the Buxco Pulmonary Function Test System (*n* = 6). D) H&E staining and lung injury scores of lung tissue from each group (*n* = 6). Scale bar: 50 µm. E) Protein concentration in BALF from mice in each group (*n* = 6). F) Lung tissue dry‐to‐wet ratio in each group (*n* = 6). G) LDH activity in lung tissue from mice in each group (*n* = 6). H) Levels of IL‐1β, IL‐6, TNF‐α, and IL‐10 in BALF (*n* = 6). I) Levels of ROS, MDA, SOD, CAT, and GSH in lung tissues (*n* = 6). J) RT‐qPCR and K) Western blot analysis of ACOD1 and Nrf2 expression in ACOD1‐overexpressing MH‐s stable cells under a high‐lipid environment, treated with ML385, and subjected to LPS stimulation (*n* = 3). L) RT‐qPCR analysis of M1 (iNOS, CD86) and M2 (Arg‐1, CD206) marker mRNA levels in MH‐s cells (*n* = 3). M) Flow cytometry was used to analyze the proportion of M1 macrophages (CD86+CD206−) and M2 macrophages (CD86−CD206+) in MH‐s cells (*n* = 3). N) Immunofluorescence detection of protein levels of M1 macrophage marker (iNOS, green) and M2 macrophage marker (Arg‐1, red) in MH‐s cells (*n* = 3). Scale bar: 20 µm. O) Western blot analysis of iNOS, CD86, Arg1, and CD206 in MH‐s cells (*n* = 3). P) Immunofluorescent labeling of ROS in MH‐s cells (*n* = 3). Scale bar: 20 µm. Data are expressed as mean ± SD.

To further investigate whether Nrf2 is a key downstream mediator of ACOD1's protective effects in exacerbated ALI in the context of obesity, we utilized the Nrf2 inhibitor ML385 in both in vivo and in vitro experiments. In macrophage‐targeted ACOD1‐overexpressing HFD mice, ML385 (30 mg k^−1 ^g) was dissolved in 10% DMSO + 40% PEG400 + 5% Tween‐80 + 45% saline and administered via intraperitoneal injection 2 h prior to LPS exposure.^[^ ^55^ ^]^ This treatment significantly inhibited Nrf2 expression despite elevated ACOD1 levels (**Figure** **7**A,B). Compared to the control group without Nrf2 inhibitor treatment, Nrf2 inhibition significantly weakened the protective effects of macrophage‐targeted ACOD1 overexpression on lung injury, as shown by deteriorated lung function (Figure 7C), worsened pathological damage (Figure 7D), increased BALF protein content, elevated W/D ratio, enhanced LDH activity (Figure 7E–G), and exacerbated inflammation and oxidative stress (Figure 7H,I; Figure S7D, Supporting Information).

In vitro experiments, we observed similar trends. In stable ACOD1‐overexpressing MH‐s cells cultured under high‐fat conditions, Nrf2 inhibition was achieved by treating the cells with ML385 (5 µm) 1 h prior to LPS exposure.^[^ ^56^, ^57^ ^]^ Despite ACOD1 overexpression (Figure 7J,K), Nrf2 inhibition markedly diminished the beneficial effects of ACOD1 on LPS‐induced inflammation and oxidative stress. This was evidenced by an increased proportion of M1 macrophages and a decreased proportion of M2 macrophages (Figure 7L–O), elevated levels of pro‐inflammatory cytokines and oxidative stress markers, and reduced anti‐inflammatory cytokines and antioxidant enzyme activities (Figure 7P; Figure S7H,I, Supporting Information).

These findings demonstrate that Nrf2 is a critical downstream mediator of ACOD1's protective role in aggravated ALI in the context of obesity. ACOD1 in macrophages mitigates LPS‐induced inflammation and oxidative stress by upregulating Nrf2 expression, thereby protecting lung tissue from injury.

### 2.8 Obesity‐Driven Upregulation of GFI1 Suppressed ACOD1 Transcription

**Figure 8:**
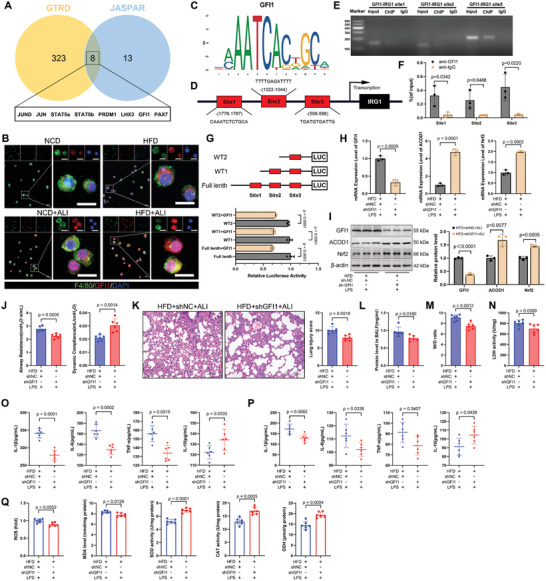
GFI1 inhibits ACOD1 transcription in the context of obesity. A) Transcription factors that can bind to the ACOD1 promoter were predicted using GTRD and JASPAR databases. B) Dual‐labeled immunofluorescence showing colocalization of GFI1(red) and F4/80(green) in primary alveolar macrophages from mice (*n* = 3). Scale bar: 20 µm. C) Motif of the transcription factor GFI1. D) Predicted GFI1 binding sites in the promoter region of IRG1. E,F) ChIP‐qPCR was performed to verify the binding relationship between GFI1 and the ACOD1 promoter. G) Dual‐luciferase reporter assay was used to confirm the regulatory relationship between GFI1 and the ACOD1 promoter. H) mRNA and I) protein expression levels of GFI1, ACOD1, and Nrf2 in the lung tissues of HFD mice with GFI1 knockdown during LPS‐induced lung injury (*n* = 3). J) Airway resistance and lung compliance in HFD mice with GFI1 knockdown during LPS‐induced lung injury (*n* = 6). K) H&E staining and lung injury scores of lung tissue in each group (*n* = 6). Scale bar: 50 µm. L) Protein concentration in BALF from mice in each group (*n* = 6). M) Lung tissue dry‐to‐wet ratio in each group (*n* = 6). N) LDH activity in lung tissue from mice in each group (*n* = 6). O) Levels of IL‐1β, IL‐6, TNF‐α, and IL‐10 in BALF (*n* = 6). P) Levels of IL‐1β, IL‐6, TNF‐α, and IL‐10 in serum (*n* = 6). Q) Levels of ROS, MDA, SOD, CAT, and GSH in lung tissues (*n* = 6). Data are expressed as mean ± SD.

To investigate the mechanism underlying the downregulation of ACOD1 in obesity, we focused on transcription factors that regulate ACOD1 expression. First, we retrieved the ACOD1 promoter sequence from the NCBI sequence database and used the JASPAR (http://jaspar.genereg.net/) and GTRD (http://gtrd.biouml.org/) databases to predict potential transcription factors involved in ACOD1 regulation. From the JASPAR database, 21 transcription factors were identified with strong correlations to ACOD1 (correlation score >500), while the GTRD database revealed 331 transcription factors. After further screening, we identified the eight transcription factors most strongly associated with ACOD1: JUND, JUN, STAT5a, STAT5b, PRDM1, LHX3, GFI1, and PAX7 (**Figure** **8**A).

We then evaluated the mRNA and protein levels of these transcription factors in the lung tissues of NCD and HFD mice. While no significant differences were observed in the mRNA levels of these factors between HFD and NCD mice (Figure S8A, Supporting Information), only GFI1 protein levels were significantly elevated in the lungs of HFD mice (LHX3 was barely detectable in lung tissue and thus omitted from the Western blot analysis) (Figure S8B, Supporting Information). Immunofluorescence staining of primary alveolar cells further demonstrated higher GFI1 expression in HFD mice compared to controls, and its nuclear translocation was more prominent following LPS exposure (Figure 8B). These findings suggest that the transcriptional repressor GFI1 is upregulated in obesity and may regulate ACOD1 transcription in lung tissue.

Chromatin immunoprecipitation (ChIP) experiments confirmed that GFI1 binds directly to the promoter region of ACOD1 (Figure 8C–F). To further delineate GFI1's functional role and the specific promoter regions involved, we performed dual‐luciferase reporter assays using truncated constructs of the ACOD1 promoter. WT1 encompassed sites 2 and 3, WT2 covered site 3, and the full‐length ACOD1 promoter was also included. The results demonstrated that GFI1 significantly inhibits ACOD1 promoter activity, with comparable inhibitory effects observed for both WT1 and WT2 relative to the full‐length promoter. These findings suggest that GFI1's inhibitory action on ACOD1 is likely independent of site 1 (Figure 8G).

To further elucidate the role of GFI1 in regulating pulmonary ACOD1 expression in the context of obesity, we specifically knocked down GFI1 expression in the lungs of HFD mice via airway administration of adeno‐associated virus (shGFI1, 5 × 10¹^2^ vg ml^−1^; 100 µl mouse^−1^, 5 × 10¹¹ vg mouse^−1^) (Figure S8C, Supporting Information). Following LPS exposure, lung function, injury, inflammation, and oxidative stress were assessed in GFI1‐knockdown HFD mice. The results showed that GFI1 knockdown significantly increased ACOD1 and its downstream target Nrf2 expression in the lungs (Figure 8H,I) and partially improved LPS‐induced lung dysfunction (Figure 8J), alleviated lung injury (Figure 8K–N), reduced inflammation (Figure 8O,P), and mitigated oxidative stress (Figure 8Q).

In summary, these findings indicate that GFI1 suppresses ACOD1 transcription in the lungs in the context of obesity, exacerbating LPS‐induced lung injury, inflammation, and oxidative stress. Airway‐targeted knockdown of GFI1 restores ACOD1 expression in the lungs and partially alleviates the worsening of LPS‐induced lung injury in obesity.

## 3 Discussion

In line with previous studies on ALI exacerbated by obesity,^[^ ^20^, ^21^, ^22^, ^23^, ^24^ ^]^ we observed that LPS exposure leads to more severe lung injury in the context of obesity. To investigate the underlying mechanisms, we identified ACOD1 as a differentially expressed gene in the lung tissue of HFD mice through sequencing analysis. Subsequent examination of lung tissue and primary alveolar macrophages from both human beings and mice confirmed that ACOD1 expression is reduced in alveolar macrophages in the context of obesity. This aligns with previous findings in hepatic macrophages and pulmonary microvascular endothelial cells,^[^ ^41^, ^58^ ^]^ indicating that ACOD1 expression may be altered in various tissues in the context of obesity. Additionally, a study reported a significant reduction of ACOD1 expression in alveolar macrophages during pulmonary fibrosis, where it plays a crucial role in disease progression.^[^ ^59^ ^]^ Since obesity has been shown to promote the development of pulmonary fibrosis,^[^ ^60^ ^]^ these observations indirectly support our finding of ACOD1 downregulation in alveolar macrophages under obesity.

To our knowledge, this study is the first to demonstrate that ACOD1 expression is downregulated in alveolar macrophages in the context of obesity, contributing to the increased severity of lung injury following LPS exposure in obese individuals. Both in vivo and in vitro experiments further suggested that this downregulation of ACOD1 in obesity may be linked to the upregulation of the transcriptional repressor GFI1 in alveolar macrophages. These findings provide new insights into the molecular mechanisms by which obesity worsens lung injury and suggest that targeting the GFI1‐ACOD1 pathway could offer potential avenues for further investigation into therapeutic strategies for obesity‐related lung complications.

As a key immunoregulatory factor, ACOD1 has been implicated in various diseases, including cardiovascular diseases,^[^ ^61^, ^62^, ^63^ ^]^ lung diseases,^[^ ^35^, ^36^, ^59^, ^64^ ^]^ cancer,^[^ ^65^, ^66^ ^]^ parasitic infections,^[^ ^67^ ^]^ and autoimmune disorders.^[^ ^68^, ^69^ ^]^ These studies consistently highlight how ACOD1 dysregulation affects inflammation and oxidative stress across different pathological contexts. In this study, we knocked down ACOD1 expression in the lungs of NCD mice to simulate the reduced levels seen in obesity, while overexpressing ACOD1 in HFD mice to reverse this downregulation. Our results showed that pulmonary ACOD1 deficiency in normal‐weight mice exacerbated LPS‐induced lung injury, while overexpression of ACOD1 in the lungs of HFD mice mitigated lung injury by providing significant anti‐inflammatory and antioxidant effects. Notably, all gene manipulations were achieved via airway administration of adeno‐associated virus (AAV), ensuring localized regulation of ACOD1 expression in the lungs and avoiding systemic immune effects as much as possible. This lung‐targeted approach allowed us to focus on local inflammatory responses.

Further experiments demonstrated that in HFD mice overexpressing ACOD1, the depletion of macrophages significantly reduced the protective effects of ACOD1, whereas neutrophil depletion had no comparable impact. This suggests that ACOD1's protective role in lung injury is primarily mediated by macrophages. However, previous studies have shown that ACOD1 acts through different effector cells depending on the disease model. For instance, in models of *Mycobacterium tuberculosis* infection and *Staphylococcus aureus* pneumonia, ACOD1 primarily exerts its effects through neutrophils,^[^ ^64^, ^70^ ^]^ while in traumatic tendon injury models, neutrophils are identified as the primary ACOD1‐expressing cells regulating local inflammation.^[^ ^71^ ^]^ These discrepancies may reflect variations in cellular activation patterns, inflammatory microenvironments, and metabolic states across different disease models. In our study, we found that, in the context of obesity, changes in ACOD1 protein and mRNA expression in alveolar macrophages were the primary factors contributing to the increased severity of lung injury. In contrast, no differences in ACOD1 expression were observed in neutrophils within our model, and their role in this context remains unclear. The observed reduction in ACOD1 expression in the context of obesity may be linked to suppressed transcription in macrophages. Subsequent in vivo experiments targeting the regulation of ACOD1 specifically in pulmonary macrophages further confirmed that ACOD1's protective effects against obesity‐aggravated lung injury are macrophage‐mediated. Given the protective role of ACOD1 within macrophages, these findings suggest the potential for developing pulmonary macrophage‐targeted therapies using biomaterial‐encapsulated ACOD1, offering new directions for future research.

ACOD1 is well known for exerting its anti‐inflammatory and antioxidant effects primarily through its catalytic product, itaconate,^[^ ^27^ ^]^ with Nrf2 widely reported as a downstream target of ACOD1.^[^ ^30^, ^41^, ^52^, ^53^, ^54^ ^]^ In our study, macrophage‐targeted overexpression of ACOD1 in an obese context restored Nrf2 expression, leading to a significant reduction in inflammation and oxidative stress. However, when an Nrf2 inhibitor was applied, the anti‐inflammatory and antioxidant effects of macrophage‐targeted ACOD1 overexpression were attenuated, confirming that Nrf2 is a key downstream mediator of ACOD1's protective effects in this model. Further analysis revealed that the protective effects of macrophage‐targeted ACOD1 overexpression were not entirely eliminated by the Nrf2 inhibitor, suggesting the involvement of other downstream pathways in the exacerbation of lung injury in the context of obesity. Although our current study did not further explore other potential mechanisms contributing to this process, this does not diminish the critical role of Nrf2 in ACOD1‐mediated protection. Investigating additional pathways that may contribute to obesity‐related lung injury could represent an important direction for future research.

Growth factor independent 1 (GFI1) is a zinc finger protein found across various organisms and acts as a transcriptional repressor.^[^ ^72^ ^]^ Previous studies have shown that GFI1 in alveolar macrophages can regulate the expression of several pro‐inflammatory cytokines in LPS‐induced ALI models.^[^ ^73^ ^]^ Additionally, obesity has been shown to significantly upregulate GFI1 protein levels in hematopoietic stem cells, contributing to obesity‐driven hematological disorders.^[^ ^74^ ^]^ In this study, we predicted GFI1's involvement using bioinformatics databases and validated it through in vivo experiments, finding that the protein level of GFI1 is notably upregulated in the lung tissue of individuals with obesity. Chromatin immunoprecipitation (ChIP) and dual‐luciferase reporter assays confirmed that GFI1, acting as a transcriptional repressor, binds to the ACOD1 promoter region and inhibits its transcription. Further animal experiments revealed that GFI1 knockdown increased ACOD1 expression and alleviated LPS‐induced inflammation in obesity, indicating the critical role of GFI1 in exacerbating lung injury. Similar to the ACOD1 manipulations, GFI1 knockdown was achieved through airway administration of AAV, ensuring lung‐specific gene regulation and avoiding systemic immune modulation.

Previous studies have shown that GFI1‐deficient mice develop severe neutropenia, and upon exposure to LPS, they succumb to uncontrolled inflammation.^[^ ^73^, ^75^ ^]^ However, in our study, GFI1 knockdown exhibited clear anti‐inflammatory and protective effects. One possible explanation is that GFI1 plays a crucial role in immune regulation, where its normal expression helps maintain immune balance. In the context of obesity, GFI1 overexpression may disrupt this balance, leading to abnormal transcriptional repression and immune dysregulation. Interestingly, we observed that the difference in GFI1 expression occurred only at the protein level, with no significant changes in transcriptional activity. This suggests that GFI1 regulation may involve post‐translational modifications, reflecting a complex regulatory mechanism in obesity. Based on existing literature, GFI1 can undergo SUMO^[^ ^76^ ^]^ and ubiquitin^[^ ^77^ ^]^ modifications, and obesity has been linked to enhanced SUMOylation.^[^ ^78^ ^]^ These findings imply the potential immune and metabolic changes in obesity that may alter GFI1 protein levels through post‐translational modifications, providing new directions for future research and enhancing our understanding of the mechanisms underlying lung injury exacerbated by obesity. While further research is needed, these insights may lay the groundwork for future studies exploring potential therapeutic strategies for managing obesity‐exacerbated lung injury.

This study has several limitations. First, the in vitro co‐culture system of alveolar macrophages and adipocytes may not fully replicate the complex in vivo microenvironment of obesity‐associated inflammation, which might affect the interpretation of results derived from these systems. To address this limitation, we performed additional experiments in vivo. After validating the role of ACOD1 in the in vitro co‐culture system. We further conducted targeted regulation of alveolar macrophages in vivo, strongly indicating that the protective effect of ACOD1 overexpression in obesity‐exacerbated acute lung injury is macrophage‐dependent. These in vivo experiments complement the in vitro findings and partially mitigate the limitations of the simplified co‐culture system. Second, we were only able to collect clinical samples from normal‐weight and patients with obesity, without including patients with ARDS. Ideally, the study would have included four groups: normal‐weight, obese, normal‐weight with ARDS, and obese with ARDS, to provide a more comprehensive assessment of the relationship between obesity and lung injury. However, ARDS patients are generally unable to undergo invasive procedures such as lobectomy or bronchoalveolar lavage, limiting our ability to obtain lung tissue and lavage samples for a full‐group analysis. We acknowledge that this limitation reduces the direct applicability of our findings to patients with obesity‐associated ARDS. To address this, we used well‐established animal models to simulate these four groups, and the results from these models largely validated our hypothesis, enhancing the generalizability of our findings. Nevertheless, it is important to recognize that while animal models provide valuable mechanistic insights, their clinical translation may be limited. Specifically, the animal experiments were confined to rodent models, which may affect the broader applicability of the results, as different species exhibit variations in physiology, metabolism, and immune responses. Large animal models may better replicate human pathophysiological processes, especially in the context of complex diseases such as lung injury. Therefore, future research should incorporate large animal models and, when feasible, include clinical samples from obese individuals with ARDS to further strengthen the clinical relevance and therapeutic potential of the study's conclusions.

## 4 Conclusion

**Figure 9:**
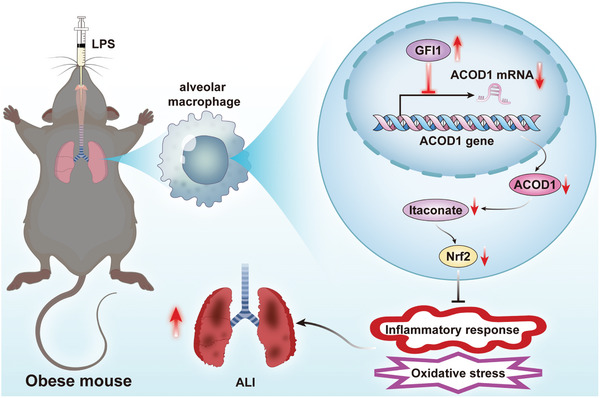
Schematic diagram of the mechanistic pathway in this study.

In conclusion, this study demonstrated that in the context of obesity, GFI1 protein levels are significantly elevated in alveolar macrophages, leading to the suppression of ACOD1 expression. This suppression exacerbates the inflammatory response and oxidative stress following LPS exposure, resulting in more severe lung injury in obese individuals (**Figure** **9**). Our findings highlight the critical protective role of ACOD1 in mitigating lung injury exacerbated by obesity and provide valuable insights for future research. While further investigation is needed, these insights may help inform the development of potential therapeutic strategies to address the heightened severity of lung injury in obesity.

## 5 Experimental Section

### Human Sample Collection

Lung tissue and bronchoalveolar lavage fluid (BALF) samples were obtained from patients at the First Affiliated Hospital of Chongqing Medical University. Lung tissue samples were collected from patients undergoing lobectomy for the diagnosis and/or treatment of solitary pulmonary nodules, and BALF samples were collected from patients undergoing gastrectomy for metabolic syndrome or gastric tumors. Informed consent was obtained from all patients, and the study was approved by the Ethics Committee of the First Affiliated Hospital of Chongqing Medical University (Approval No: 2023–326). Patients were grouped based on body mass index (BMI) into the obesity group (BMI ≥ 30 kg m^−^^2^) and the normal weight group (BMI ≥ 18.5 kg m^−^^2^ to <25 kg m^−^^2^). Lung tissue samples were collected at least 5 cm away from the pulmonary nodule,^[^ ^79^ ^]^ and all patients had solitary pulmonary nodules (≤2 cm) detected during routine exams with no recent lung infections. Exclusion criteria included underlying pulmonary diseases (asthma, COPD, interstitial lung disease, ARDS, or cor pulmonale), lung infection, smoking history, autoimmune diseases, patient refusal, and findings of invasive malignancy on intraoperative frozen biopsy. BALF samples were collected from patients undergoing sleeve gastrectomy or gastric tumor resection. These patients had either metabolic syndrome or gastric tumors detected during routine physical examinations, and none had recent lung infections. Exclusion criteria for BALF sampling were pre‐existing pulmonary diseases, lung infection, smoking history, autoimmune diseases, intraoperative hypoxemia, and patient refusal. Intraoperative findings of invasive malignancy also led to exclusion. Detailed patient information is provided in Table S1 (Supporting Information).

### Animal Study

Eight‐week‐old male C57BL/6J mice were used, and the study protocol was approved by the Animal Ethics Committee of Chongqing Medical University (IACUC‐CQMU‐2023‐0062). The mice (five per cage) were housed under pathogen‐free conditions at 24 ± 1 °C with a 12‐hour light/dark cycle. They were randomly divided into two groups: a normal chow diet (NCD) group and a high‐fat diet (HFD) group (60% fat content, TP2330055A, Trophic Animal Feed High‐tech Co., Ltd., China) and fed for 16 weeks. Mice were anesthetized with 2.5% isoflurane, and 100 µL of LPS (1 mg mL^−1^, L2630, Sigma‐Aldrich, St. Louis, MO, USA) was administered intratracheally to induce lung injury, while the control group received an equal volume of sterile saline. Assessments were conducted 24 h after administration.

### Pulmonary Function Testing in Mice

Lung function in mice was assessed using the Buxco Pulmonary Function Testing (PFT) system (DSI Buxco, St. Paul, MN, USA), calibrated and configured with the required parameters. Mice were anesthetized via intraperitoneal injection of ketamine (50 mg k^−1 ^g) and medetomidine (0.33 mg k^−1 ^g). After a tracheotomy was performed, a blunt 19‐gauge needle was inserted and secured with sutures to prevent air leakage. The mice were then placed in the PFT chamber and connected to a ventilator. Airway resistance (RI) and dynamic lung compliance (Cdyn) were measured in Resistance and Compliance (RC) mode using FinePointe PFT software and Buxco PFT controller hardware. Data were recorded every 2 s after baseline stabilization, and an average was calculated from 2 min of data collection per mouse.

### Collection and Culture of Primary Alveolar Macrophages

Primary alveolar macrophages were isolated from the bronchoalveolar lavage fluid (BALF) of both humans and mice. For human samples, following intubation under general anesthesia, lung lavage was performed with a total of 60 mL of sterile saline at 37 °C, instilled in 20 mL increments over three rounds. BALF was recovered via a fiberoptic bronchoscope with negative pressure suction, ensuring a total recovery rate of over 30%. For mice, after euthanasia, the trachea was exposed, and an 18G catheter was inserted and secured. The lungs were lavaged 10 times, each time with cold PBS (1 mL) containing EDTA (0.5 mm), pausing for 1 min before aspirating the BALF.

The collected BALF was centrifuged at 1500 rpm for 10 min at 4 °C, and the cell pellet was resuspended in RPMI‐1640 medium supplemented with 10% fetal bovine serum (FBS), 100 U mL^−1^ penicillin, and 100 µg mL^−1^ streptomycin. The cell suspension was plated in 6‐well plates and allowed to adhere for 1–3 h. Non‐adherent cells were removed by washing twice with PBS. Lung tissue remaining after lavage was not used for further studies.

### Cell Culture

MH‐s murine alveolar macrophages (ATCC, Manassas, VA, USA) were cultured in RPMI‐1640 medium (Gibco, Waltham, MA, USA) supplemented with 10% FBS and 1% penicillin‐streptomycin, and incubated at 37 °C with 5% CO₂. Cells were stimulated with 100 ng mL^−1^ LPS (L2630, Sigma‐Aldrich, St. Louis, MO, USA) for 24 h.

3T3‐L1 mouse embryonic fibroblasts were obtained from Procell Life Science and Technology (Wuhan, China) and cultured in DMEM with 10% FBS and 1% penicillin‐streptomycin until confluence. Differentiation was induced using DMEM containing 10% FBS, 1 µm dexamethasone, 0.5 mm IBMX, and 10 µg mL^−1^ insulin for 3 days, followed by maintenance in DMEM with 10% FBS and 10 µg mL^−1^ insulin for 4 days, with media changes every 2 days. After differentiation, cells were maintained in DMEM with 10% FBS.

Differentiated 3T3‐L1 cells were seeded in the lower chamber of a Transwell system, while MH‐s cells were seeded in the upper chamber with 0.4 µm pores. The two cell types were co‐cultured at 37 °C with 5% CO₂ for 48 h. After co‐culture, 100 ng mL^−1^ LPS was added to the MH‐s cells, and 24 h later, inflammatory cytokine expression in the culture supernatant, macrophage polarization, and oxidative stress status were assessed.

### Lentivirus and Adeno‐Associated Virus (AAV)

MH‐s cells were infected with lentiviruses to stably knock down ACOD1 (shACOD1) (LV‐H1‐shRNA‐ACOD1‐PURO) or overexpress ACOD1 (LvACOD1) (LV‐CMV‐ACOD1‐PURO).

In mice, adeno‐associated viruses (AAV) were administered via airway delivery to achieve specific genetic modifications in the lungs. including stable knockdown of ACOD1 (shACOD1, AAV6‐U6‐shRNA‐ACOD1), overexpression of ACOD1 (AAV‐ACOD1, AAV6‐CMV‐ACOD1), macrophage‐specific knockdown of ACOD1 (AAV‐F4/80‐shACOD1, AAV9‐F4/80p‐shRNA‐ACOD1), macrophage‐specific overexpression of ACOD1 (AAV‐F4/80‐ACOD1, AAV9‐F4/80p‐ACOD1), and knockdown of GFI1 (shGFI1, AAV6‐U6‐shRNA‐GFI1), following the manufacturer's instructions.

The lentiviruses and AAVs were constructed with assistance from Hanbio (Shanghai, China), GenePharma (Shanghai, China), and GeneChem (Shanghai, China). The target sequence for ACOD1 knockdown was 5′‐CCAGCAGGATGTGGCCT TTAA‐3′, the overexpression sequence was NM_0 08392.1, and the target sequence for GFI1 knockdown was 5′‐GCTCCTACA AATGCATCAAAT‐3′.

### Itaconate Detection

Lung tissue was treated with pre‐chilled 50% methanol, sonicated at 4 °C for 30 min, and then centrifuged at 13 000 r min^−1^ for 15 min. The supernatant was filtered through a 0.22 µm membrane and analyzed using an ultra‐performance liquid chromatography (UPLC) system (Waters, Milford, MA, USA) equipped with an HSS T3 column (2.1 × 100 mm, 1.8 µm; Waters, Milford, MA, USA). The flow rate was maintained at 0.3 mL min^−1^, and the column temperature was set to 30 °C. Gradient elution was performed using mobile phase A (ultrapure water) and mobile phase B (acetonitrile). The separated compounds were detected using an AB Sciex 4000 QTRAP MS/MS system (AB Sciex, Framingham, MA, USA) in negative ion electrospray ionization (ESI) mode, targeting the itaconate ion pair at m/z 128.9/84.8. The concentration of itaconate in the samples was quantified using a standard curve, with adjustments for sample dilution.

### Oil Red O Staining

Following the method described in previous studies,^[^ ^80^ ^]^ heart, liver, kidney, and lung tissues were rapidly frozen in liquid nitrogen and embedded in OCT. Fresh frozen sections were prepared, fixed, and washed with distilled water. After air drying, the sections were stained for 15 min using the Oil Red O Staining Kit (G1260, Solarbio, Beijing, China). The sections were then differentiated, counterstained with hematoxylin for 1 min, rinsed with distilled water, and mounted for further analysis.

### Hematoxylin and Eosin (H&E) Staining and Lung Injury Scoring

The right lower lung lobe was fixed in 4% paraformaldehyde (G1101, Servicebio, Wuhan, China) for 24 h, embedded in paraffin, and sectioned at a thickness of 5 µm. Tissue sections were stained with H&E following standard protocols. Lung injury severity was evaluated using a semi‐quantitative scoring system as described in previous studies.^[^ ^81^ ^]^ Briefly, four indicators were assessed: alveolar septal thickening, inflammation, hemorrhage, and edema. Blinded scoring was conducted on randomly selected fields from each section (*n* = 3 group^−1^). The scoring criteria for alveolar septal thickening, hemorrhage, and edema were as follows: none (0), mild (1), moderate (2), severe (3), and very severe (4). Inflammation was evaluated by counting the total number of inflammatory cells per ×100 field.

### Lung Wet/Dry (W/D) Weight Ratio

The right upper lung lobe was weighed to determine the wet weight (*W*), following previously described methods.^[^ ^82^ ^]^ The tissue was then dehydrated at 65 °C for 72 h, and the dry weight (*D*) was recorded.

### Bronchoalveolar Lavage Fluid (BALF) Collection and Analysis

After euthanizing the mice, the right main bronchus was ligated, and 0.5 mL of sterile saline was instilled into the left lung three times. The collected BALF was centrifuged at 1500 rpm for 10 min at 4 °C, and the supernatant was stored at −80 °C for further analysis. Protein concentration in the BALF was measured using a bicinchoninic acid (BCA) protein assay kit (P0010, Beyotime, Shanghai, China).

### Measurement of ROS

Reactive oxygen species (ROS) levels in lung tissue and MH‐s cells were measured using 2′,7′‐dichlorofluorescin diacetate (DCFH‐DA) (E‐BC‐K138‐F, Elabscience, Wuhan, China). Single‐cell suspensions were prepared, incubated with 10 µm DCFH‐DA at 37 °C for 30 min, and washed three times with PBS. ROS levels in lung tissue were quantified using a fluorescence microplate reader (TECAN, Männedorf, Switzerland) at 500 nm excitation and 525 nm emission wavelengths, while ROS generation in MH‐s cells was visualized using a confocal microscope (ZEISS, Oberkochen, Germany).

### Measurement of MDA, SOD, CAT, and GSH

Lung tissue or cell homogenates were lysed in extraction buffer, and the levels of MDA, SOD, CAT, and GSH were measured using the corresponding assay kits, following the manufacturer's instructions: MDA (S0131S, Beyotime, Shanghai, China), SOD (S0101, Beyotime, Shanghai, China), CAT (S0051, Beyotime, Shanghai, China), and GSH (A006, Nanjing Jiancheng Bioengineering Institute, Nanjing, China).

### Biochemical Analysis

Lung lactate dehydrogenase (LDH) activity was measured using a commercial kit (BC0685, Solarbio, Beijing, China).

Pro‐inflammatory cytokine levels (IL‐6, IL‐1β, IL‐10, and TNF‐α) in BALF, serum, and cell culture supernatants were determined using ELISA kits (GEM0001, GEM0002, GEM0003, GEM0004, Servicebio, Wuhan, China).

Serum triglyceride (TG) and total cholesterol (TC) levels were measured with commercial kits (A110‐1‐1, A111‐1‐1, Nanjing Jiancheng Bioengineering Institute, Nanjing, China).

Fasting blood glucose and glucose tolerance were assessed using glucose test strips and a glucometer (Sinocare Biosensor Co., Ltd., China). Mice were fasted overnight (12 h) and then subjected to an intraperitoneal glucose tolerance test (IPGTT) by injecting glucose solution (2 g kg^−1^, 200 mg mL^−1^) intraperitoneally. Blood glucose levels were measured at 0, 30, 60, 90, and 120 min after glucose administration.

### Flow Cytometry Analysis

Cells were scraped from culture dishes and washed three times with cold PBS. Macrophage polarization was assessed using PE‐conjugated anti‐mouse CD86 (12‐0862‐82, Thermo Fisher Scientific, Waltham, MA, USA) and APC‐conjugated anti‐mouse CD206 (17‐2061‐82, Thermo Fisher Scientific, Waltham, MA, USA) antibodies, according to the manufacturer's instructions. Multicolor flow cytometry was then performed, and data were acquired and analyzed using CytExpert software (version 10.0.7; Tree Star, Ashland, OR, USA).

### Chromatin Immunoprecipitation (ChIP)

ChIP assays were performed on MH‐s cells using a ChIP Kit (BW2501, Guangzhou Baiwei Biotechnology, Guangzhou, China), following the manufacturer's instructions. Briefly, 2–5 × 10⁷ MH‐s cells were cross‐linked with 1% formaldehyde by adding 37% formaldehyde, with the reaction quenched by glycine. Cells were collected, washed with PBS, and lysed on ice to extract nuclei. DNA was sheared into 200–600 bp fragments using sonication. The lysate was incubated with magnetic beads pre‐bound to GFI1 antibody or IgG antibody (negative control) to enrich DNA bound to the target protein. After multiple washes to remove nonspecific proteins and DNA, the target DNA was eluted from the antibody‐bead complexes at 65 °C, followed by reverse cross‐linking to remove the proteins. The purified DNA was extracted using phenol‐chloroform and precipitated with ethanol. The enriched DNA fragments were then analyzed by qPCR to assess the strength of protein‐DNA interactions. Primers for the GFI1‐ACOD1 binding site are listed in Table S3 (Supporting Information).

### Dual‐Luciferase Reporter Assay

The full‐length ACOD1 gene, ACOD1‐WT1, and ACOD1‐WT2 were cloned into luciferase reporter plasmids and co‐transfected with GFI1 and the Renilla control plasmid (pRL‐TK) into MH‐s cells (gene sequences are listed in Table S2, Supporting Information). After 48 h, the cells were lysed, and luciferase activity was measured using the Dual‐Luciferase Reporter Assay System (Promega, San Luis Obispo, CA, USA).

### Quantitative Real‐Time PCR (qRT‐PCR)

Total RNA from lung tissue and cells was extracted using TRIzol reagent (9108, Takara Biotechnology, Dalian, China) according to the manufacturer's instructions. Reverse transcription was performed using the Evo M‐MLV RT Mix Kit (AG11728, Accurate Biology, Changsha, China), followed by qPCR using the SYBR Green Premix Pro Taq HS qPCR Kit (AG11701, Accurate Biology, Changsha, China). Relative mRNA expression levels were normalized to β‐Actin and calculated using the 2*^−ΔΔCt^* method. All primer sequences used in this study are listed in Table S3 (Supporting Information).

### Immunofluorescence

Paraffin‐embedded lung tissue sections were deparaffinized, rehydrated, and subjected to antigen retrieval using sodium citrate solution (P0081, Beyotime, Shanghai, China). MH‐s cells were fixed with 4% paraformaldehyde and permeabilized with 0.1% Triton X‐100 (ST797, Beyotime, Shanghai, China). Both tissues and cells were blocked with 10% fetal bovine serum (C0234, Beyotime, Shanghai, China) for 1 h at room temperature to prevent nonspecific binding. Slides were incubated overnight at 4 °C with specific primary antibodies, followed by a 1‐hour incubation with fluorescent secondary antibodies (1:200, Proteintech, Wuhan, China) at room temperature. Slides were mounted with an anti‐fade mounting medium containing DAPI (P0131, Beyotime, Shanghai, China), and images were captured after 10 min. Fluorescence in tissue sections was imaged using an upright fluorescence microscope (Nikon, Tokyo, Japan), and cell fluorescence was captured using a confocal microscope (ZEISS, Oberkochen, Germany). All primary antibodies used in this study are listed in Table S4 (Supporting Information).

### Western Blot

Cells or tissues were lysed in RIPA buffer (G2002, Servicebio, Wuhan, China) containing 1% phenylmethylsulfonyl fluoride (PMSF, G2008, Servicebio, Wuhan, China). The lysates were subjected to 10–15% SDS‐polyacrylamide gradient gel (SDS‐PAGE) electrophoresis for protein separation and then transferred onto PVDF membranes. Membranes were blocked with 5% non‐fat milk to prevent nonspecific binding and incubated with primary antibodies at 4 °C for 12 h. Afterward, they were incubated with HRP‐conjugated secondary antibodies (anti‐rabbit or anti‐mouse, 1:3000, Proteintech, Wuhan, China) for 2 h at 37 °C. β‐Actin served as the loading control. Protein bands were visualized using an enhanced chemiluminescence (ECL) system (Bio‐Rad, Hercules, CA, USA), and relative protein expression levels were quantified with ImageJ software (Bethesda, MD, USA). All primary antibodies used in this study are listed in Table S4 (Supporting Information).

### Statistical Analysis

Data were presented as mean ± standard deviation (mean ± SD). Statistical analyses were conducted using GraphPad Prism 9.5 (GraphPad Software, San Diego, CA, USA). A two‐tailed unpaired Student's *t*‐test was used for comparisons between two groups, while one‐way ANOVA followed by Tukey's post hoc test was applied for multiple group comparisons. Statistical significance was defined as *p* < 0.05 for all analyses.

## Conflict of Interest

The authors declare no conflict of interest.

## Supporting information



Supporting Information

## Data Availability

The data that support the findings of this study are available from the corresponding author upon reasonable request.
