# Supplementary material for: Elevated GFI1 in Alveolar Macrophages Suppresses ACOD1 Expression and Exacerbates Lipopolysaccharide‐Induced Lung Injury in Obesity
Source: Adv Sci (Weinh). 2025 Feb 8;12(13):2413546. doi: 10.1002/advs.202413546 (PMC11967830; doi:10.1002/advs.202413546)
Supplement: Supplementary file 1 — Supporting Information [file ADVS-12-2413546-s001.pdf]

ADVANCED SCIENCE
Open Access

Supporting Information

for *Adv. Sci.*, DOI 10.1002/advs.202413546

Elevated GFI1 in Alveolar Macrophages Suppresses ACOD1 Expression and Exacerbates Lipopolysaccharide-Induced Lung Injury in Obesity

Jingyue Ma, Yichan Ao, Zhen Yue, Zhiqiao Wang, Xiangyu Hou, Hongbin Li, Hanbing Wang, Siqing Luo, Jianyu He, Zikun Duan, Ling Liu and Ke Wei**

Supporting Information

Elevated GFI1 in Alveolar Macrophages Suppresses ACOD1 Expression and Exacerbates Lipopolysaccharide-Induced Lung Injury in Obesity

Jingyue Ma, Yichan Ao, Zhen Yue, Zhiqiao Wang, Xiangyu Hou, Hongbin Li, Hanbing Wang, Siqing Luo, Jianyu He, Zikun Duan, Ling Liu, Ke Wei**

Figure S1.

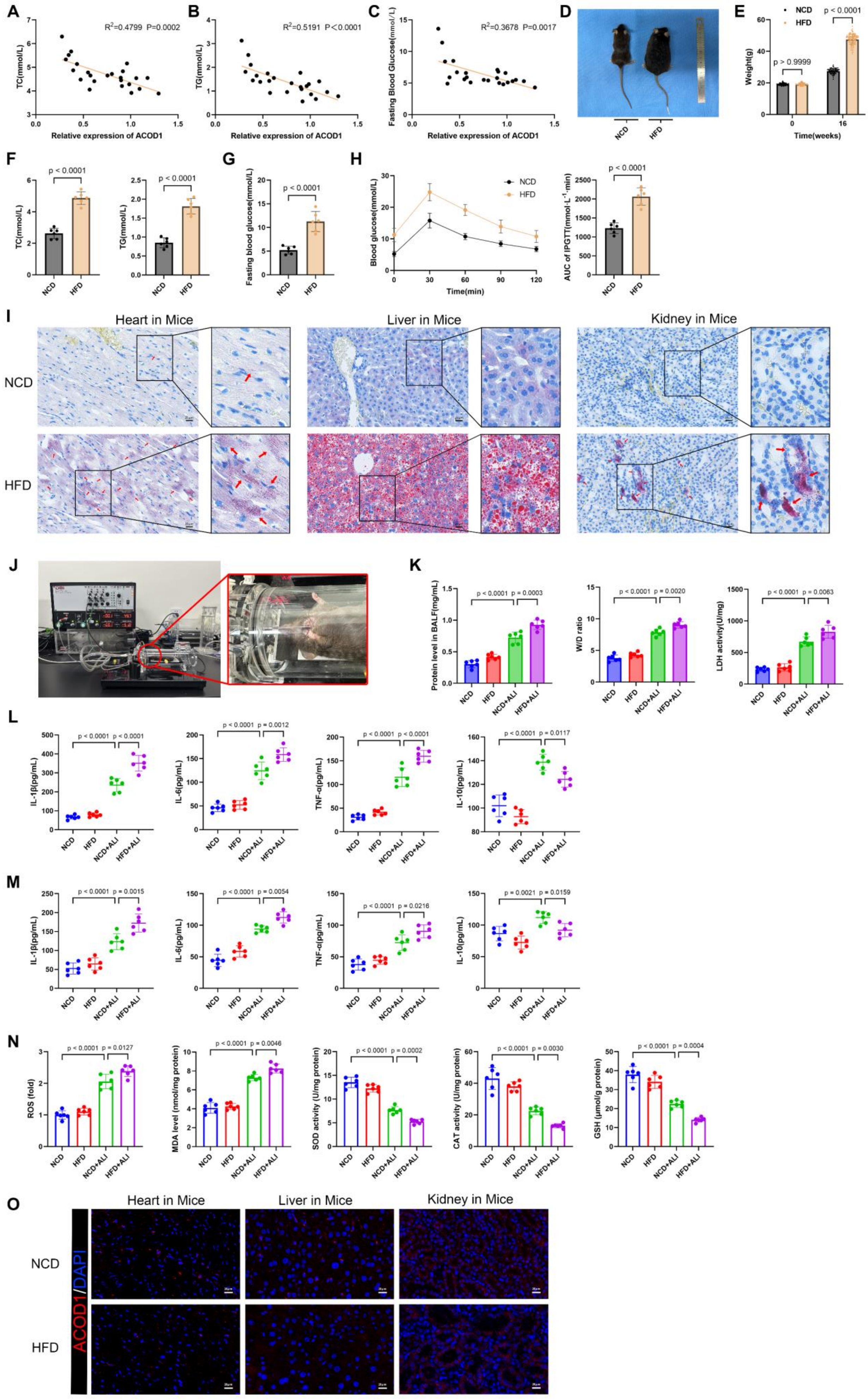

A
B
C
D
E
F
G
H
I
J
K
L
M
N
O
Relative expression of ACOD1
TC(mmol/L)
TG(mmol/L)
Fasting Blood Glucose(mmol/L)
R2=0.4799 P=0.0002
R2=0.5191 P<0.0001
R2=0.3678 P=0.0017
NCD
HFD
Weight(g)
Time(weeks)
p > 0.9999
p < 0.0001
Fasting blood glucose(mmol/L)
Blood glucose(mmol/L)
Time(min)
AUC of IPGTT(mmol·L-1·min)
Heart in Mice
Liver in Mice
Kidney in Mice
Protein level in BALF(mg/mL)
W/D ratio
LDH activity(U/mg)
NCD+ALI
HFD+ALI
IL-1β(pg/mL)
IL-6(pg/mL)
TNF-α(pg/mL)
IL-10(pg/mL)
ROS (fold)
MDA level (nmol/mg protein)
SOD activity (U/mg protein)
CAT activity (U/mg protein)
GSH (μmol/g protein)
ACOD1/DAPI

Figure S1: Reduction of ACOD1 in lung tissue in the context of obesity. A) Correlation between ACOD1 expression levels and total cholesterol (TC) in human lung tissue. B) Correlation between ACOD1 expression levels and triglyceride (TG) in human lung tissue. C) Correlation between ACOD1 expression levels and fasting blood glucose in human lung tissue. D) Representative images of NCD and HFD mice. E) Body weight changes over 16 weeks in NCD (n=75) and HFD (n=135) mice. F) Serum lipid levels (TC, TG) in NCD and HFD mice (n=6). G) Fasting blood glucose levels in NCD and HFD mice (n=6). H) Changes in blood glucose levels during IPGTT in NCD and HFD mice, with area under the curve (AUC) analysis of IPGTT results (n=6). I) Oil red O staining of heart, liver, and kidney tissues from NCD and HFD mice, with red arrows indicating typical lipid droplets (n=3). Scale bar: 20 μm. J) Buxco Pulmonary Function Testing (PFT) system for assessing lung function in mice. K) Protein concentration in BALF, lung dry-to-wet weight ratio, and LDH activity in lung tissue from NCD and HFD mice 24 hours after LPS treatment (n=6). L)Levels of IL-1β, IL-6, TNF-α, and IL-10 in BALF of mice (n=6). M) Levels of IL-1β, IL-6, TNF-α, and IL-10 in serum of mice (n=6). N) Levels of ROS, MDA, SOD, CAT, and GSH in lung tissues of mice (n=6). O) Immunofluorescence staining of ACOD1 in heart, liver, and kidney tissues from mice (n=3). Scale bar: 20 μm. Data are expressed as mean ± SD.

Figure S2.

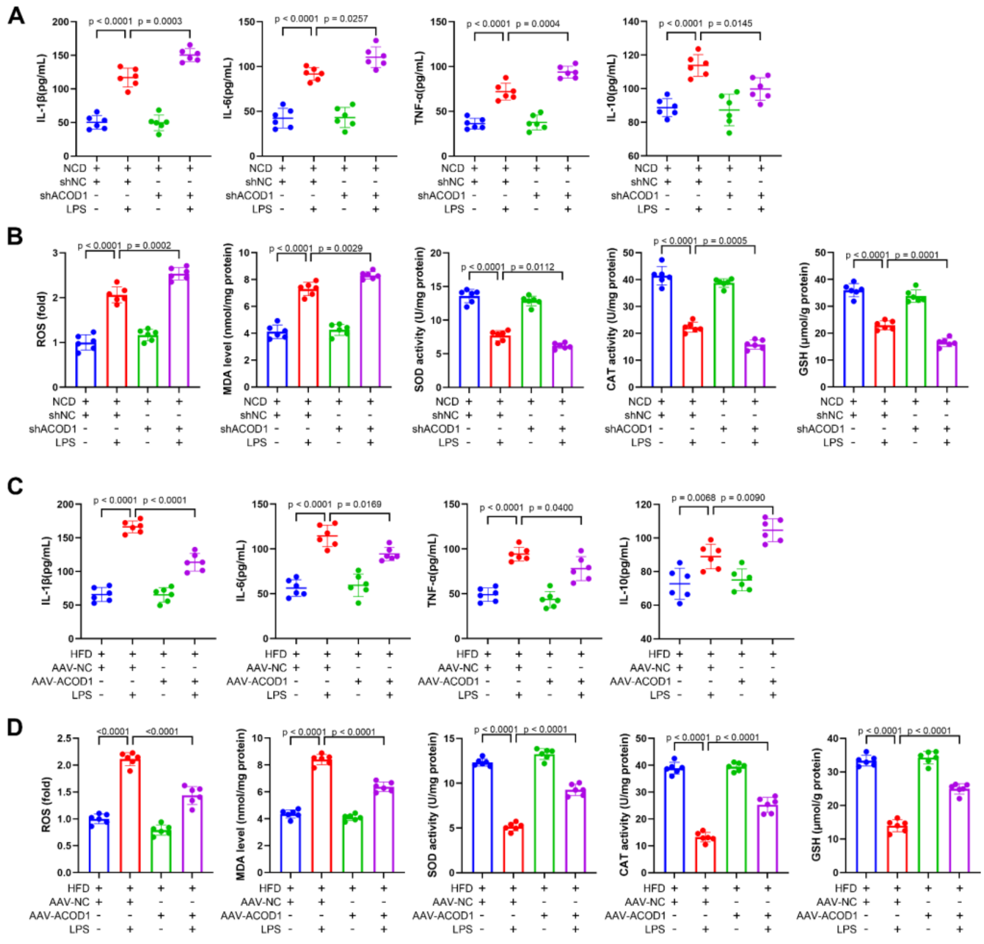

Figure S2: Regulation of Lung ACOD1 Modulates LPS-Induced Lung Injury. A) Levels of IL-1β, IL-6, TNF-α, and IL-10 in serum of NCD mice (n=6). B) Levels of ROS, MDA, SOD, CAT, and GSH in lung tissues of NCD mice (n=6). C) Levels of IL-1β, IL-6, TNF-α, and IL-10 in serum of HFD mice (n=6). B) Levels of ROS, MDA, SOD, CAT, and GSH in lung tissues of HFD mice (n=6). Data are expressed as mean ± SD.

Figure S3.

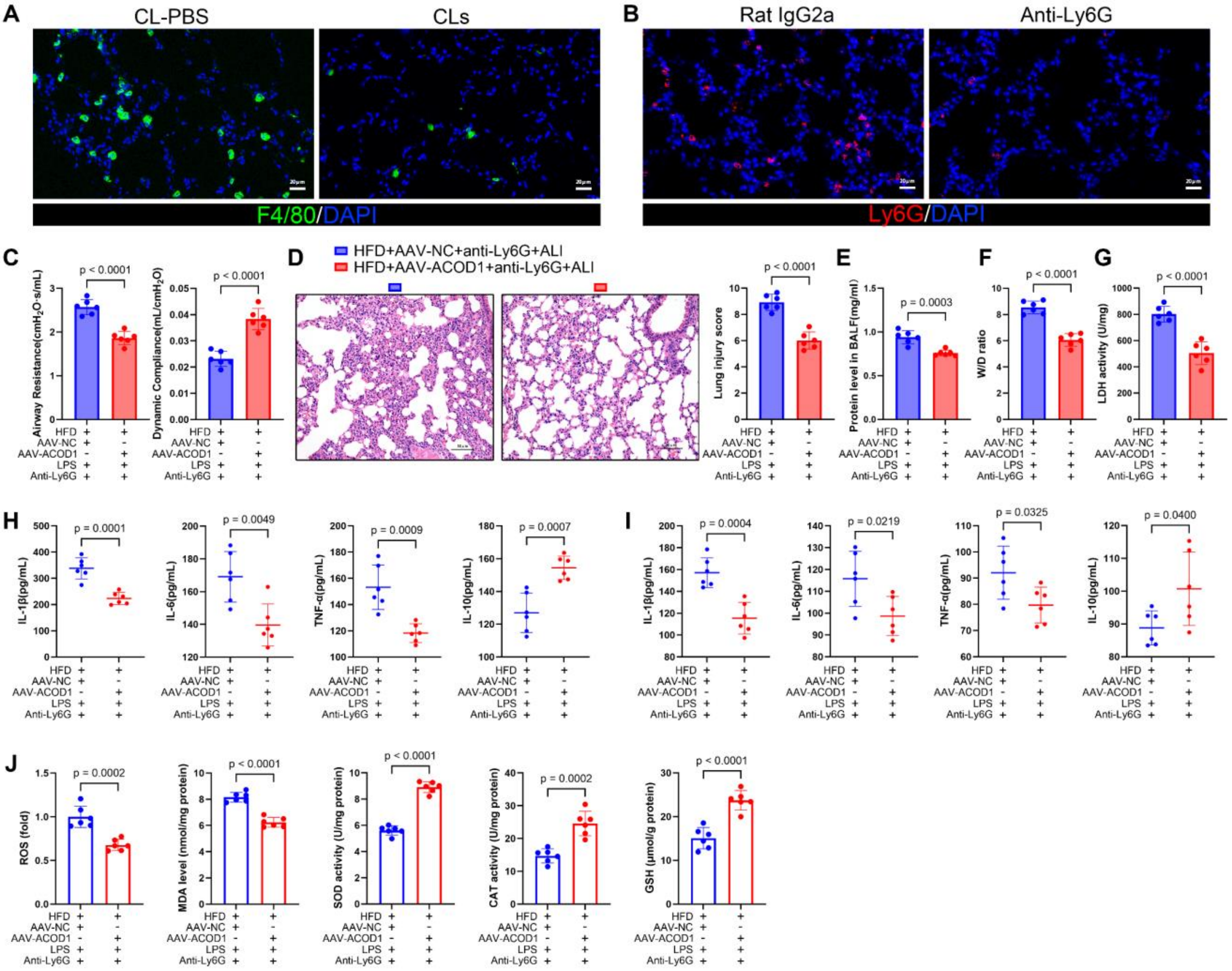

Figure S3: Alveolar macrophages play a major role in ACOD1-mediated protection against ALI in obesity. A) Immunofluorescence of macrophages in mouse lung tissue, F4/80 (green), DAPI (blue). Scale bar: 20 μm. B) Immunofluorescence of neutrophils in mouse lung tissue, Ly6G (red), DAPI (blue). Scale bar: 20 μm. C) Airway resistance and lung compliance in different groups after neutrophil depletion(n=6). D) H&E staining and lung injury score in lung tissue from each group (n=6). Scale bar: 50 μm. E) Protein concentration in BALF from each group (n=6). F) Lung tissue dry-to-wet ratio in each group (n=6). G) LDH activity in lung tissue from each group (n=6). H) Levels of IL-1β, IL-6, TNF-α, and IL-10 in BALF were measured by ELISA (n=6). I) Levels of IL-1β, IL-6, TNF-α, and IL-10 in serum were measured by ELISA

(n=6). J) Levels of ROS, MDA, SOD, CAT, and GSH in lung tissue were measured using commercial kits (n=6). Data are expressed as mean ± SD.

Figure S4.

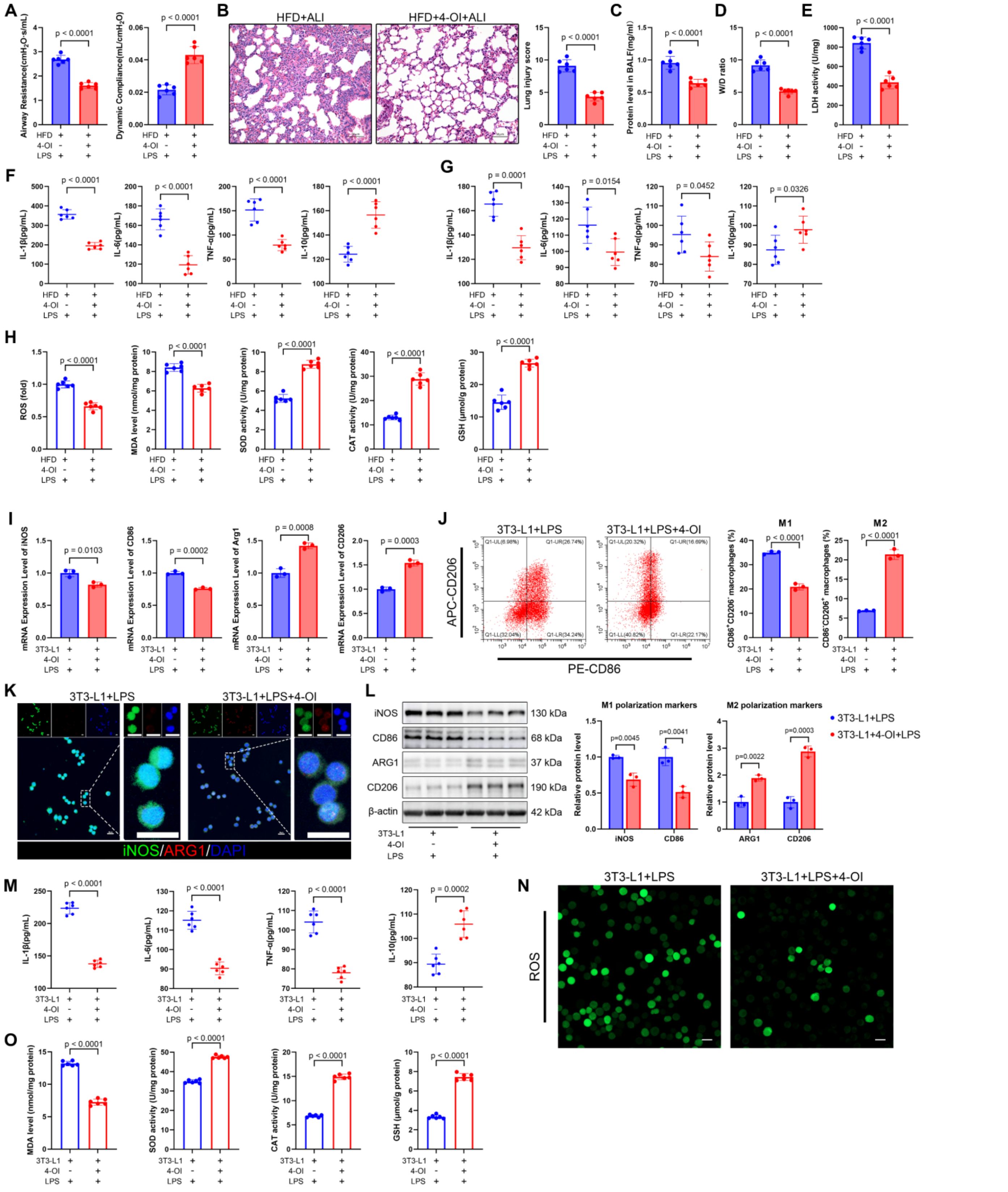

Figure S4: 4-OI alleviates LPS-induced lung injury and macrophage inflammation and oxidative stress in HFD mice. A) Airway resistance and lung compliance in HFD mice treated with 4-OI during LPS-induced

ALI(n=6). B) H&E staining and lung injury scores of lung tissue in each group (n=6). Scale bar: 50 μm. C) Protein concentration in BALF in each group (n=6). D) Lung tissue dry-to-wet ratio in each group (n=6). E) LDH activity in lung tissue in each group (n=6). F) Levels of IL-1β, IL-6, TNF-α, and IL-10 in BALF(n=6). G) Levels of IL-1β, IL-6, TNF-α, and IL-10 in serum(n=6). H) Levels of ROS, MDA, SOD, CAT, and GSH in lung tissues(n=6). I) RT-qPCR analysis of M1 (iNOS, CD86) and M2 (Arg-1, CD206) markers in MH-s cells under a high-lipid environment, treated with 4-OI, and exposed to LPS(n=3). J) Flow cytometry analysis of the proportion of M1 macrophages (CD86+CD206−) and M2 macrophages (CD86−CD206+) in MH-s cells across groups (n=3). K) Immunofluorescence detection of protein levels of M1 macrophage marker (iNOS, green) and M2 macrophage marker (Arg-1, red) in MH-s cells(n=3). Scale bar: 20 μm. L) Western blot analysis of iNOS, CD86, Arg1, and CD206 in MH-s cells under a high-lipid environment, treated with 4-OI, and exposed to LPS(n=3). M) levels of IL-1β, IL-6, TNF-α, and IL-10 in the supernatant of cell culture medium(n=6). N) Immunofluorescent labeling of ROS in MH-s cells under a high-lipid environment, treated with 4-OI, and exposed to LPS (n=3). Scale bar: 20 μm. O) Levels of MDA, SOD, CAT, and GSH in MH-s cells(n=6). Data are expressed as mean ± SD.

Figure S5.

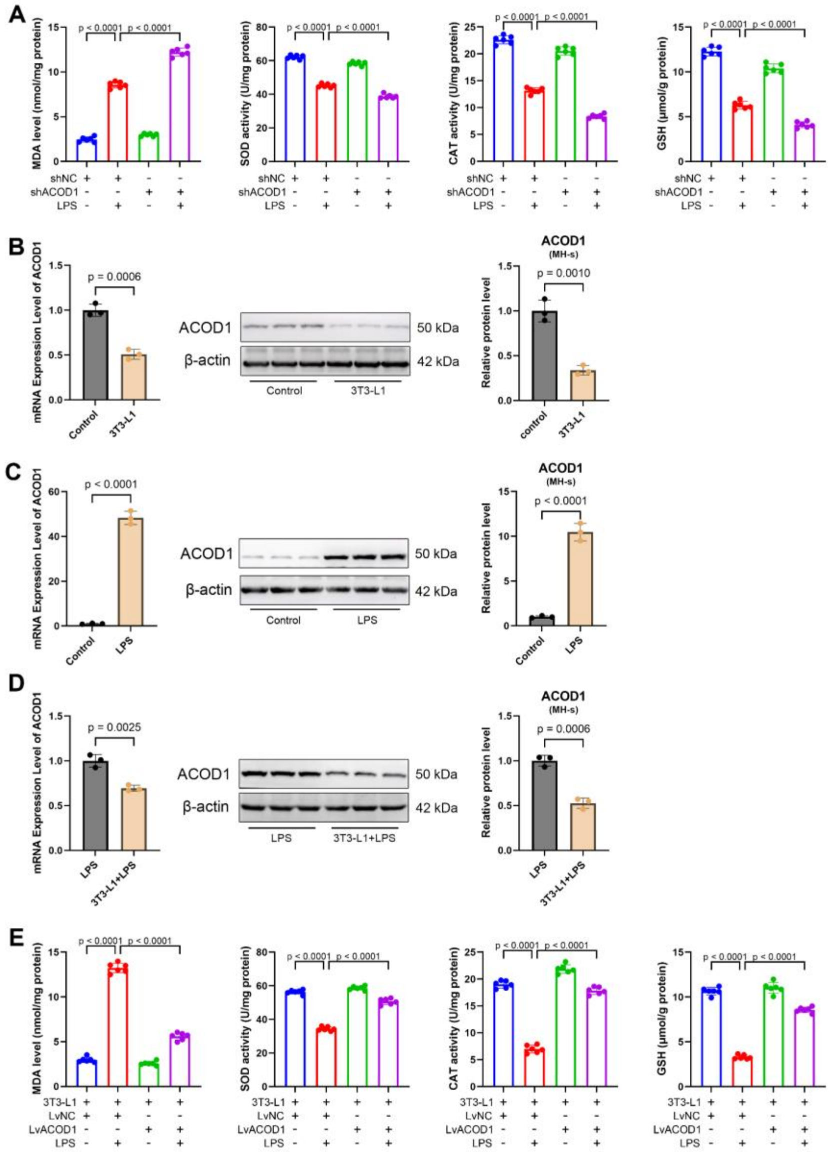

Figure S5: Regulation of ACOD1 in MH-s Modulates LPS-Induced Inflammation and Oxidative Stress.

A) Levels of MDA, SOD, CAT and GSH in ACOD1-knockdown MH-s cells(n=6). B) RT-qPCR and Western blot analysis of ACOD1 expression in MH-s cells under a high-lipid environment(n=3). C) RT-qPCR and Western blot analysis of ACOD1 expression in MH-s cells following LPS stimulation(n=3). D) RT-qPCR and Western blot analysis of ACOD1 expression in MH-s cells under a high-lipid environment following LPS stimulation(n=3). E) Levels of MDA, SOD, CAT, and GSH in ACOD1-overexpressing MH-s cells under a high-lipid environment (n=6). Data are expressed as mean ± SD.

Figure S6.

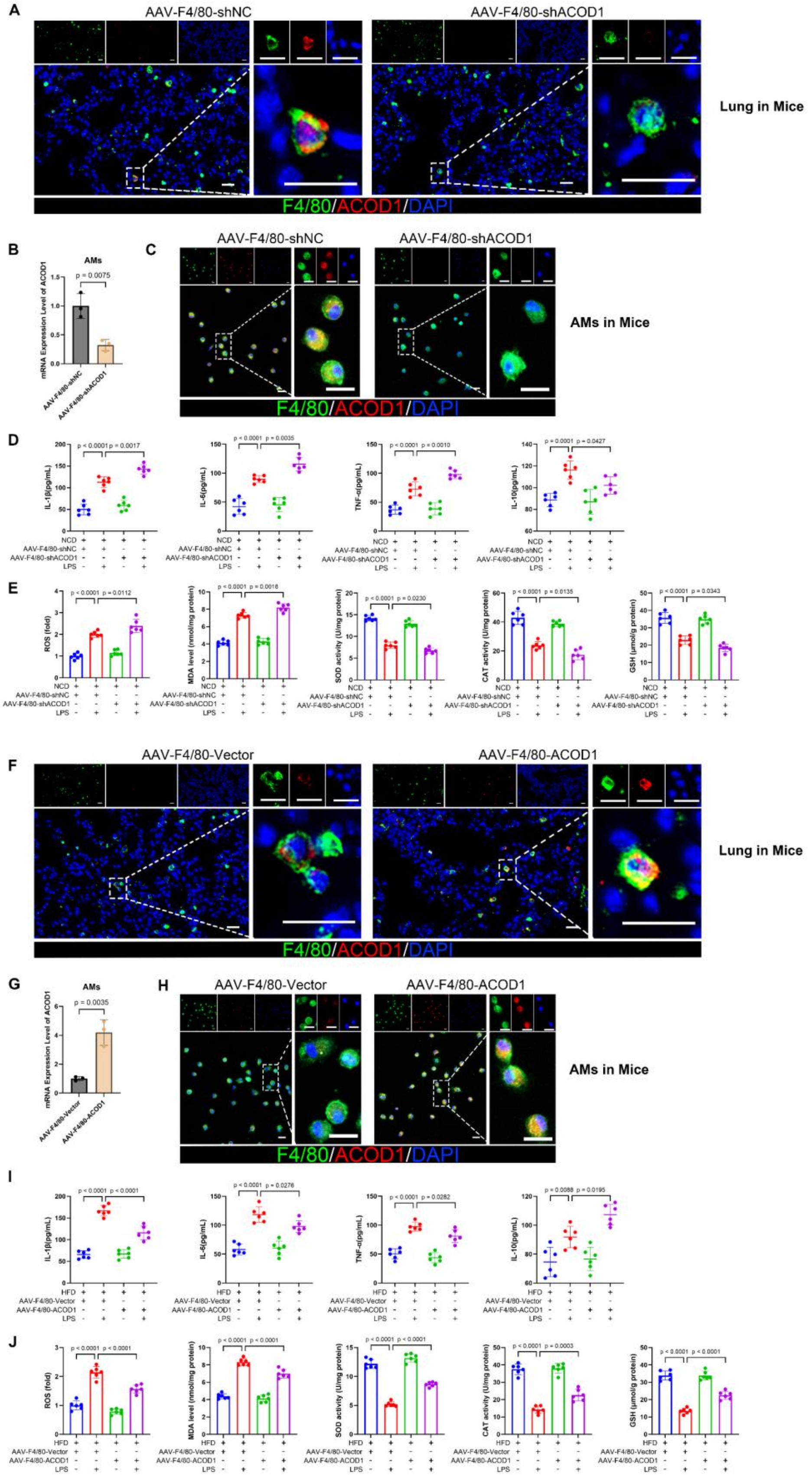
A
AAV-F4/80-shNC
AAV-F4/80-shACOD1
Lung in Mice
F4/80/ACOD1/DAPI
B
AMs
p = 0.0075
C
AAV-F4/80-shNC
AAV-F4/80-shACOD1
AMs in Mice
F4/80/ACOD1/DAPI
D
E
F
AAV-F4/80-Vector
AAV-F4/80-ACOD1
Lung in Mice
F4/80/ACOD1/DAPI
G
AMs
p = 0.0035
H
AAV-F4/80-Vector
AAV-F4/80-ACOD1
AMs in Mice
F4/80/ACOD1/DAPI
I
J

Figure S6: Targeted regulation of macrophage ACOD1 modulates LPS-induced lung injury. A) Dual-labeled immunofluorescence staining analysis ACOD1 (red) expression in macrophages (F4/80, green) in mouse lung tissue 4 weeks after airway administration of AAV-F4/80-shACOD1(n=3). Scale bar: 20 μm. B) RT-qPCR analysis of ACOD1 mRNA levels in primary alveolar macrophages from mice 4 weeks after airway administration of AAV-F4/80-shACOD1(n=3). C) Immunofluorescence analysis of ACOD1 (red) in primary alveolar macrophages (F4/80, green) from mice 4 weeks after airway administration of AAV-F4/80-shACOD1(n=3). Scale bar: 20 μm. D) Levels of IL-1β, IL-6, TNF-α, and IL-10 in serum of NCD mice with lung macrophage-specific ACOD1 knockdown (n=6). E) Levels of ROS, MDA, SOD, CAT, and GSH in lung tissues of NCD mice with lung macrophage-specific ACOD1 knockdown (n=6). F) Dual-labeled immunofluorescence staining analysis ACOD1 (red) expression in macrophages (F4/80, green) in mouse lung tissue 4 weeks after airway administration of AAV-F4/80-ACOD1(n=3). Scale bar: 20 μm. G) RT-qPCR analysis of ACOD1 mRNA levels in primary alveolar macrophages from mice 4 weeks after airway administration of AAV-F4/80-ACOD1(n=3). H) Immunofluorescence analysis of ACOD1 (red) in primary alveolar macrophages (F4/80, green) from mice 4 weeks after airway administration of AAV-F4/80-ACOD1(n=3). Scale bar: 20 μm. I) Levels of IL-1β, IL-6, TNF-α, and IL-10 in serum of HFD mice with lung macrophage-specific ACOD1 overexpression (n=6). J) Levels of ROS, MDA, SOD, CAT, and GSH in lung tissues of HFD mice with lung macrophage-specific ACOD1 overexpression (n=6). Data are expressed as mean ± SD.

Figure S7.

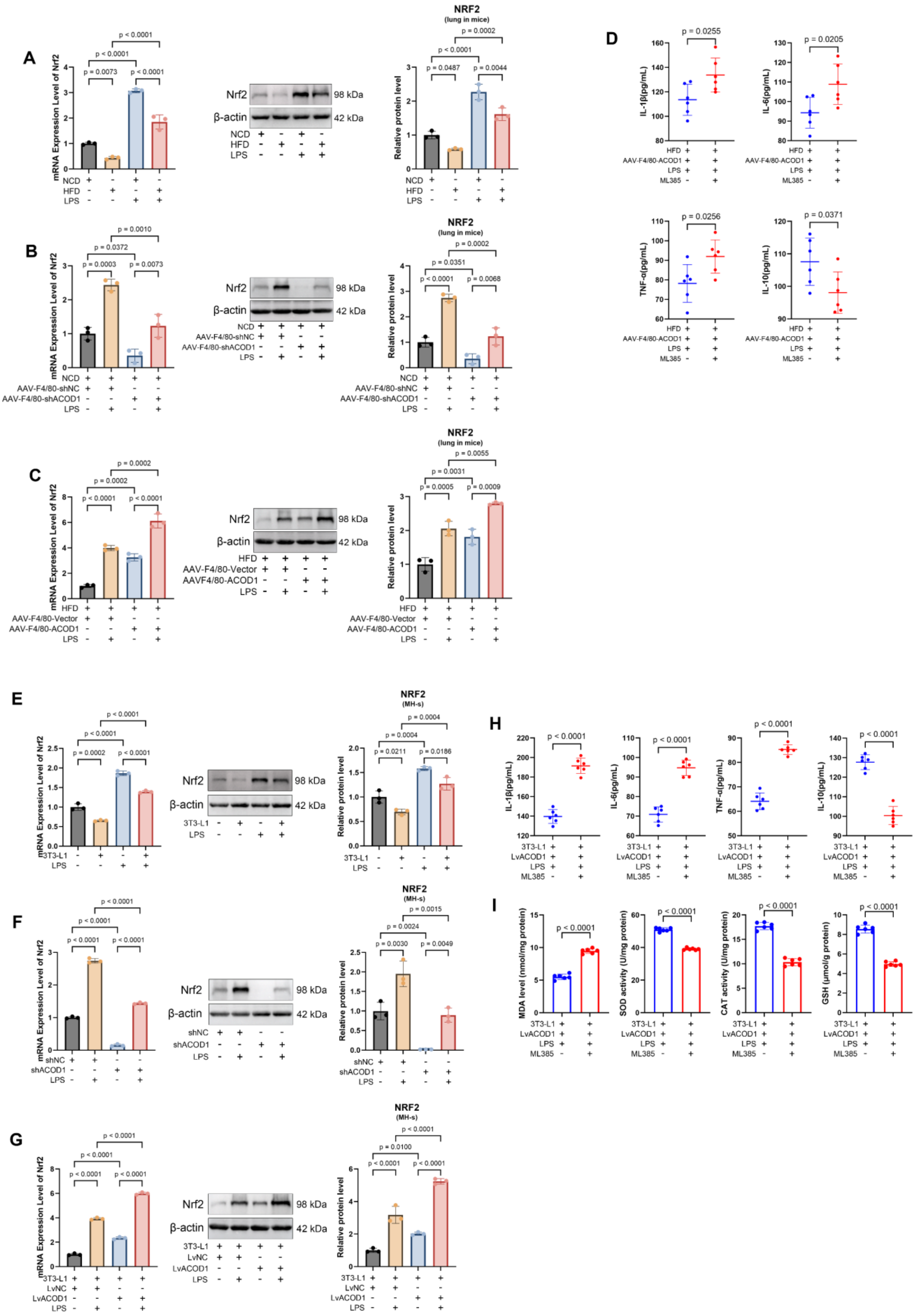
A
mRNA Expression Level of Nrf2
p < 0.0001
p = 0.0073
p < 0.0001
NCD
HFD
LPS
Nrf2
98 kDa
β-actin
42 kDa
NRF2
(lung in mice)
p = 0.0002
p = 0.0487
p = 0.0044
Relative protein level
B
p = 0.0010
p = 0.0372
p = 0.0003
p = 0.0073
AAV-F4/80-shNC
AAV-F4/80-shACOD1
p = 0.0351
p < 0.0001
p = 0.0068
C
p = 0.0002
p < 0.0001
AAV-F4/80-Vector
AAV-F4/80-ACOD1
AAVF4/80-ACOD1
p = 0.0055
p = 0.0031
p = 0.0005
p = 0.0009
D
p = 0.0255
IL-1β(pg/mL)
p = 0.0205
IL-6(pg/mL)
ML385
p = 0.0256
TNF-α(pg/mL)
p = 0.0371
IL-10(pg/mL)
E
3T3-L1
NRF2
(MH-s)
p = 0.0004
p = 0.0211
p = 0.0186
F
shNC
shACOD1
p = 0.0015
p = 0.0024
p = 0.0030
p = 0.0049
G
LvNC
LvACOD1
p = 0.0100
H
I
MDA level (nmol/mg protein)
SOD activity (U/mg protein)
CAT activity (U/mg protein)
GSH (μmol/g protein)

Figure S7: Nrf2 plays an important role in ACOD1-mediated ALI protection in obesity. A-C) mRNA and protein expression levels of Nrf2 in lung tissues from mice in each group (n=3). D) Serum levels of IL-1β, IL-6, TNF-α, and IL-10 in HFD mice with lung macrophage-specific ACOD1 overexpression treated with ML385 and LPS (n=6). E-G) mRNA and protein expression levels of Nrf2 in MH-s cells (n=3). H) Levels of IL-1β, IL-6, TNF-α, and IL-10 in the supernatant of ACOD1-overexpressing MH-s cells cultured in a high-lipid environment and treated with ML385 and LPS(n=6). I) Levels of MDA, SOD, CAT, and GSH in MH-s cells(n=6). Data are expressed as mean ± SD.

Figure S8.

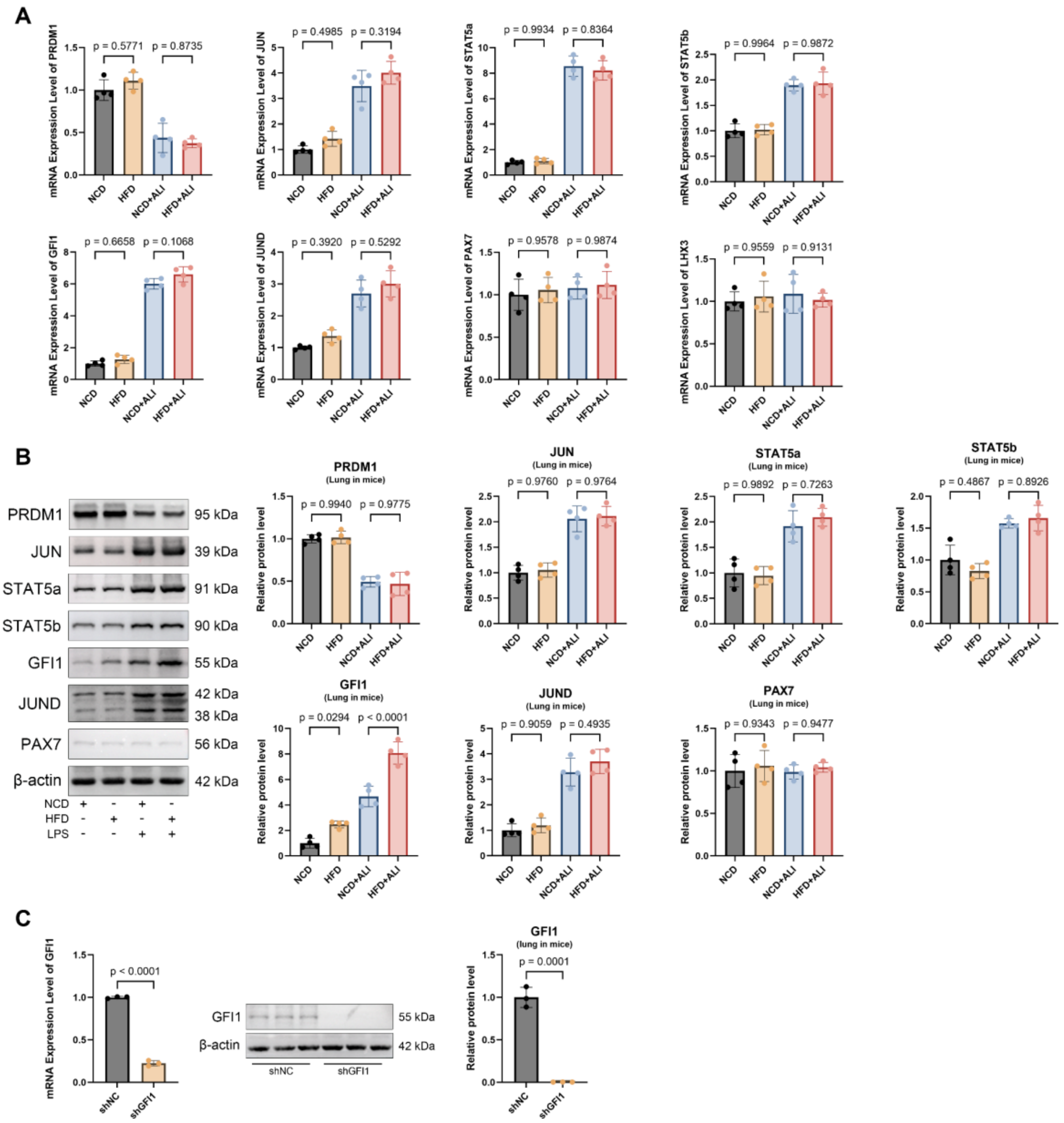

Figure S8: GFI1 inhibits ACOD1 transcription in the context of obesity. A) mRNA expression levels of JUND, JUN, STAT5a, STAT5b, PRDM1, LHX3, GFI1, and PAX7 in mouse lung tissues were measured by RT-qPCR (n=4). B) Protein expression levels of JUND, JUN, STAT5a, STAT5b, PRDM1, GFI1, and PAX7 in mouse lung tissues were measured by Western blot (n=4). **C)** mRNA and protein expression levels of GFI1 in mouse lung tissue 4 weeks after infection with adeno-associated virus shGFI1 (n=3). Data are expressed as mean ± SD.

Table S1. Patient Demographics & Clinical Characteristics

	**Lung Tissue**			**Bronchoalveolear Lavage Fluid(BALF)**		
	Lean Group (BMI 18.5-24.9 kg/m²)	Obese Group (BMI≥30.0 kg/m²)	P-value	Lean Group (BMI 18.5-24.9 kg/m²)	Obese Group (BMI≥30.0 kg/m²)	P-value
Number of Patients	12	12		5	5	
Age (years)	51.6 ± 6.708	50.2 ± 5.132	0.567	35.8 ± 3.194	31.2 ± 6.221	0.180
Gender	6 Males / 6 Females	5 Males / 7 Females		3 Males / 2 Females	3 Males / 2 Females	
BMI (kg/m²)	22.6 ± 1.492	33.9 ± 3.577	< 0.0001	22.3 ± 0.891	47.7 ± 6.693	< 0.0001
PaO_2(mmHg) (before induction FiO_2 21%)	84 ± 4	83 ± 4	0.203	86 ± 3	83 ± 6	0.228
$PaCO_2$(mmHg) (before induction FiO_2 21%)	40 ± 3	42 ± 3	0.177	39 ± 3	41 ± 2	0.193
pH (before induction)	7.39 ± 0.04	7.39 ± 0.03	0.814	7.42 ± 0.04	7.41 ± 0.05	0.779
WBC count (10^9 /L)	5.99 ± 1.46	6.27 ± 1.43	0.633	7.12 ± 1.04	7.09 ± 0.61	0.954
NEUT (%)	52.7 ± 5.72	53.6 ± 6.71	0.727	54.5 ± 6.32	54.5 ± 6.15	0.988
AST(U/L)	21.4 ± 5.3	20.2 ± 5.5	0.576	21.2 ± 3.1	23.8 ± 2.8	0.201
ALT(U/L)	18.4 ± 7.0	19.1 ± 6.1	0.805	21.2 ± 4.7	34.6 ± 19.3	0.171
Serum creatinine（μmol/L）	55.9 ± 9.6	57.1 ± 8.1	0.750	56.8 ± 13.0	63.2 ± 6.4	0.352
Blood urea nitrogen (mmol/L)	5.7 ± 1.3	5.9 ± 1.0	0.775	5.6 ± 1.1	5.8 ± 1.1	0.850
Glucose (mmol/L)	5.6 ± 0.8	7.3 ± 2.7	0.043	5.1 ± 0.3	7.4 ± 2.1	0.040
TC（mmol/L）	4.43 ± 0.38	4.99 ± 0.63	0.016	4.26 ± 0.35	5.49 ± 0.93	0.024
TG（mmol/L）	1.09 ± 0.35	1.72 ± 0.63	0.006	1.39 ± 0.22	2.94 ± 1.42	0.042
HDL（mmol/L）	1.25 ± 0.20	1.08 ± 0.17	0.037	1.25 ± 0.08	1.00 ± 0.20	0.035

LDL (mmol/L)	3.34 ± 0.47	3.82 ± 0.50	0.024	3.31 ± 0.45	3.91 ± 0.25	0.031

Data are expressed as the mean ± SD.

Table S2. Gene sequences for Dual-luciferase reporter assay used in this study.

	Nucleotide sequence
**GFI1-CDS**	ATGCCGCGCTCATTCCTGGTCAAGAGCAAGAAGGCGCACAGCTATCACCAGCCGCGTTCTCCGGGGCC GGACTACTCCCTGCGCCTGGAGACCGTGCCTGCGCCGGGCAGAGCAGAGGGCGGCGCTGTGAGTGCA GGCGAGTCGAAAATGGAGCCCCGAGAGCGTTTGTCCCCCGACTCTCAGCTTACCGAGGCTCCCGACAG GGCCTCCGCGTCCCCCAACAGCTGCGAAGGCAGCGTTTGTGACCCCTGCTCCGAGTTCGAGGACTTTT GGAGGCCCCCTTCTCCCTCCGTGTCTCCAGCGTCGGAGAAGTCACTGTGCCGCTCTCTGGACGAAGCC CAGCCCTACACGCTGCCTTTCAAGCCCTATGCATGGAGCGGTCTTGCTGGGTCTGACCTGCGGCACCTG GTGCAGAGCTATCGGCAGTGCAGCGCGCTGGAGCGCAGCGCGGGCCTGAGCCTCTTCTGCGAGCGCGG CTCGGAGCCGGGCCGCCCGGCAGCGCGCTACGGCCCCGAGCAGGCTGCGGGCGGAGCCGGTGCGGGA CAGCCAGGGAGCTGCGGGGTCGCCGGGGGCGCCACCAGCGCTGCGGGCCTGGGGCTCTACGGCGACT TCGCGCCTGCGGCGGCCGGGCTGTACGAGCGGCCGAGCACAGCAGCAGGCCGGCTGTACCAAGATCAT GGCCACGAGCTGCACGCGGACAAGAGCGTGGGCGTCAAGGTGGAGTCGGAGCTGCTTTGCACCCGTC TGCTGCTGGGCGGCGGCTCCTACAAATGCATCAAATGCAGCAAGGTGTTCTCCACACCGCACGGGCTG GAGGTGCACGTGCGCCGGTCCCACAGCGGCACAAGACCCTTTGCGTGCGAGATGTGCGGCAAGACCTT CGGGCACGCGGTGAGCCTGGAGCAACACAAGGCAGTGCACTCCCAGGAACGCAGCTTTGACTGTAAG ATCTGTGGCAAGAGCTTCAAGAGGTCATCCACGCTGTCCACACATCTGCTCATTCACTCGGACACCCGG CCCTATCCCTGTCAGTACTGTGGCAAAAGATTCCACCAGAAGTCAGATATGAAGAAACACACCTTCATC CACACAGGTGAGAAGCCCCACAAATGCCAGGTGTGCGGCAAAGCCTTCAGTCAGAGCTCCAACCTCAT CACTCATAGCAGAAAGCACACAGGCTTCAAGCCCTTTGGCTGTGACCTGTGTGGGAAGGGCTTCCAGA GGAAGGTGGATCTCAGGAGGCACCGAGAGACTCAGCATGGACTCAAATGA
**ACOD1-Full Length**	ATAAGTTACAAGTGTTACAGCCCTGTGATCTCACCAGGCTTCAGGGGGACACACAAAAACTGTTGGTAA GTATTACAGTTTGAACGCTGTTAGGGGCTTTGGAGTGCTAGCCAATAGCAATCCCTGGCAGAACAATTCT GCAGAGTGATCATTGACAGGGGTAACAAGCCTAGCATACCTCCTGTCTCTGGCAGGTCTTGAAGGCTTG AGTTGCAAATCTCTGCAAGTCTCTGAAGCAATGAATCTTGGGTAGTCCAAGTAGTTGCATGAATGAATTA CCTTCTGAATTCAAAATATGAAGCTTTGTGGACTGAAGGAAGATGAGTCCCCCAACCTGCAGCTGCAGA AAGAGCCTGTAGACTGCTCTGAGCACATTCTCTGCCCGGGACAGAAGCCTCCAGTGTGGCAAGACTCT TAACTAGTCTGTCTCCTCTCTGAAAACTTAAAGGCATTCTTCTTTCTCAGGAGAAAGTCTGGGAGCCAA GCTCCTGGTCAGACAGGGATCCAGCTGAGAGCAGGACTTCATCTTTAGGCCTCTTCTTCTGCAAAAAAG GGGAACATGTGAGGGCCAGGACACAGGTCAGAACCAGAAGACTTCAGAGAGTCAGAATCCTGCCTGC CTGGGTGCCTGGTTCTGAAGTCCTCCTCCTCCTCCTCCTCCTCCTCCTCCTCCTCCTCCTCCTCT TCCTCCTCCTCCTTCTTTCTCTCTCTCTCTCTCTCTCTCTCTCTCTCTCTCTCTCTCTCTCTCTCTC TCTCTCTCTTCTCCAAGGAATAATCTGTGAAGGATGATTTTCCAGAGTTCTCCTCTATTACACTCAACCA GGTGGTCTCTGATTAGCCAAATGTGTGTACTTGGCATGTGTGTGTGTGTGTGTGTGTGTGTGTGTGTG TGTGTGTGTACATGTGTCTGTGTGGTGTGTATTTAGATATTTTTAATTAATTTTTTGAGATTTTAATTTATTT ATTTATGTATTTACAACATTTCCACCCCTCCTCCTTCTCTTCTTTTAACTCTGGCCACCATTCCTCACAAAT

	TCATGGCCTCTTCTTTAGTTATCACACACACACACACACACACACACACACACACACACGTGTGCACAC<br>ATATGTACACGTACTTTCTCAACTGTCTCTGAGCCTTAGGTGTAGGGGTTTCATTGTTGGTATATCAGTTA<br>GGTCTGGGCACCCATGCTCACTTACTCGTTATAGATAGTTGTAGTAGTCGTCTCCATCTACAATAAAAAG<br>AAGCATCGTTGATGAGGGGCAGAGCTACACTTATCTGTGCGTGAGTAAGTATTTAGAATGCAGCTAGGT<br>TTAGGGAACTGGCAGTGGTAGGCTCTCCTCTAGGATTCATAACCTTTAGGTTATGGCACATTTAGTGACT<br>TCGTTAGTTACCTCTGGGTTATCGCCTCTGAAATTATGATGTGATTGGTGGCTTTGTTCACAGTACCAGTC<br>ATGGATTTCCTTCTGGTAAGCAGGCTCTAAGTCCAATAAGAACCAGTCAGGGTTTTGTTTTTTGTTTTT<br>TTCATCATTCCCAGAAGCTGTTTCATGGTTGGTTGGTGACAATGGGCTGTCTGTGAGAAGGCTGCTCTG<br>AGTGACTCCTTGCCTTTCCAGATCTGGGGGCTTTTCTTCCTCTGTCATAGTCTTGCTATTTCCTTGTGATA<br>AGTCTTCAAGAGTGGGATGCCCAGTAAAGAGAGTGTGCACTCTTCTCACCTATGGCCCCTAGCTGGGAC<br>AGGCCATCCCTCTGTAATTATAACTTAGTTTTTCTTATGTCTGTGAGGAATCATCAGATTATGGCCGCTGT<br>AACACCTCCTCTCATCAGCCCTACATCTATAAGGGAGCTCTACACAACACAACAAAGGAAGTTTAAGTG<br>AGGTCATTGGGGAGGTGTTGAAATGGTGGAAATTCCATGGTTGAAACAAAAGTGAAAGGGACAGACCT<br>GGAGGGAGTGACTGTGTATAAAGGCACACGTCCACTAAAGGCCC
**ACOD1-WT1**	GGATCCAGCTGAGAGCAGGACTTCATCTTTAGGCCTCTTCTTCTGCAAAAAAGGGGAACATGTGAGGG<br>CCAGGACACAGGTCAGAACCAGAAGACTTCAGAGAGTCAGAATCCTGCCTGCCTGGGTGCCTGGTTCT<br>GAAGTCCTCCTCCTCCTCCTCCTCCTCCTCCTCCTCCTCCTCCTCCTCTTCCTCCTCCTTCT<br>TTCTCTCTCTCTCTCTCTCTCTCTCTCTCTCTCTCTCTCTCTCTCTCTCTCTCTCTCTCTCTCTCTTCTCCA<br>AGGAATAATCTGTGAAGGATGATTTTCCAGAGTTCTCCTCTATTACACTCAACCAGGTGGTCTCTGATTA<br>GCCAAATGTGTGTACTTGGCATGTGTGTGTGTGTGTGTGTGTGTGTGTGTGTGTGTGTGTGTACATGT<br>GTCTGTGTGGTGTGTATTTAGATATTTTTAATTAATTTTTTGAGATTTTAATTTATTTATTTATGTATTTACAA<br>CATTTCCACCCCTCCTCCTTCTCTTCTTTTAACTCTGGCCACCATTCCTCACAAATTCATGGCCTCTTCTT<br>TAGTTATCACACACACACACACACACACACACACACACACACACGTGTGCACACATATGTACACGTACT<br>TTCTCAACTGTCTCTGAGCCTTAGGTGTAGGGGTTTCATTGTTGGTATATCAGTTAGGTCTGGGCACCCA<br>TGCTCACTTACTCGTTATAGATAGTTGTAGTAGTCGTCTCCATCTACAATAAAAAGAAGCATCGTTGATGA<br>GGGGCAGAGCTACACTTATCTGTGCGTGAGTAAGTATTTAGAATGCAGCTAGGTTTAGGGAACTGGCAG<br>TGGTAGGCTCTCCTCTAGGATTCATAACCTTTAGGTTATGGCACATTTAGTGACTTCGTTAGTTACCTCTG<br>GGTTATCGCCTCTGAAATTATGATGTGATTGGTGGCTTTGTTCACAGTACCAGTCATGGATTTCCTTCTGG<br>TAAGCAGGCTCTAAGTCCAATAAGAACCAGTCAGGGTTTTGTTTTTTGTTTTTTTCATCATTCCCAGAA<br>GCTGTTTCATGGTTGGTTGGTGACAATGGGCTGTCTGTGAGAAGGCTGCTCTGAGTGACTCCTTGCCTT<br>TCCAGATCTGGGGGCTTTTCTTCCTCTGTCATAGTCTTGCTATTTCCTTGTGATAAGTCTTCAAGAGTGGG<br>ATGCCCAGTAAAGAGAGTGTGCACTCTTCTCACCTATGGCCCCTAGCTGGGACAGGCCATCCCTCTGTA<br>ATTATAACTTAGTTTTTCTTATGTCTGTGAGGAATCATCAGATTATGGCCGCTGTAACACCTCCTCTCATC<br>AGCCCTACATCTATAAGGGAGCTCTACACAACACAACAAAGGAAGTTTAAGTGAGGTCATTGGGGAGG<br>TGTTGAAATGGTGGAAATTCCATGGTTGAAACAAAAGTGAAAGGGACAGACCTGGAGGGAGTGACTG<br>TGTATAAAGGCACACGTCCACTAAAGGCCC
**ACOD1-WT2**	CCATGCTCACTTACTCGTTATAGATAGTTGTAGTAGTCGTCTCCATCTACAATAAAAAGAAGCATCGTTGA<br>TGAGGGGCAGAGCTACACTTATCTGTGCGTGAGTAAGTATTTAGAATGCAGCTAGGTTTAGGGAACTGG<br>CAGTGGTAGGCTCTCCTCTAGGATTCATAACCTTTAGGTTATGGCACATTTAGTGACTTCGTTAGTTACCT<br>CTGGGTTATCGCCTCTGAAATTATGATGTGATTGGTGGCTTTGTTCACAGTACCAGTCATGGATTTCCTTC<br>TGGTAAGCAGGCTCTAAGTCCAATAAGAACCAGTCAGGGTTTTGTTTTTTGTTTTTTTCATCATTCCCA<br>GAAGCTGTTTCATGGTTGGTTGGTGACAATGGGCTGTCTGTGAGAAGGCTGCTCTGAGTGACTCCTTGC<br>CTTTCCAGATCTGGGGGCTTTTCTTCCTCTGTCATAGTCTTGCTATTTCCTTGTGATAAGTCTTCAAGAGT

	GGGATGCCCAGTAAAGAGAGTGTGCACTCTTCTCACCTATGGCCCCTAGCTGGGACAGGCCATCCCTCT GTAATTATAACTTAGTTTTCTTATGTCTGTGAGGAATCATCAGATTATGGCCGCTGTAACACCTCCTCTC ATCAGCCCTACATCTATAAGGGAGCTCTACACAACACAACAAAGGAAGTTTAAGTGAGGTCATTGGGGA GGTGTTGAAATGGTGGAAATTCCATGGTTGAAACAAAAGTGAAAGGGACAGACCTGGAGGGAGTGAC TGTGTATAAAGGCACACGTCCACTAAAGGCCC

Table S3. Primers sequences for qRT-PCR used in this study.

	Gene	Sequences (5'-3')	
**Mouse**	Gfi1-Irg1 site1	F: TCCTGTCTCTGGCAGGTCTT	R: GTTGGGGGACTCATCTTCCT
	Gfi1-Irg1 site2	F: CATGTGTCTGTGTGGTGTGT	R: ATTTGTGAGGAATGGTGGCC
	Gfi1-Irg1 site3	F: ACCTCTGGGTTATCGCCTCT	R: CTGGAAAGGCAAGGAGTCAC
	β-actin	F: AGATTACTGCTCTGGCTCCTAGC	R: ACTCATCGTACTCCTGCTTGCT
	iNOS	F: CAAGCTGAACTTGAGCGAGGA	R: TTTACTCAGTGCCAGAAGCTGGA
	CD86	F: ATCCAAGAGCCACTCCTACCT	R: TCCAGACCTTTCCAGGCATTT
	Arg-1	F: GCATATCTGCCAAAGACATCGT	R: CCATCACCTTGCCAATCCC
	CD206	F: AGAGCTGGCGAGCATCAAGAG	R: TTCCATAGGTCAGTCCCAACCAA
	ACOD1	F: TTCAGGCTCCCACCGACATA	R: GCTGTGAGGACAGGTAGGAC
	Nrf2	F: TATCTCCTAGTTCTCCGCTGCTC	R: GTGGCAACTCCAAGTCCATCAT
	GFI1	F: TTTGCGTGCGAGATGTGC	R: GACAGCGTGGATGACCTCTTG
	JUND	F: CTTGGGCTGCTCAAACTCG	R: GGTAGAGGAACTGCGTACTGGTC
	JUN	F: ACGACCTTCTACGACGATGC	R: GCCAGGTTCAAGGTCATGCT
	STAT5a	F: ACACAGCTCCAGAACACGTA	R: GTCAACCAGGACACCAGCAG
	STAT5b	F: CCTGCTGTCCAGTCTAAGCC	R: CAGCCACCCTGAAGACACAT
	PRDM1	F: CATACCGAAGGGAACACGCT	R: ATTCACGTAGCGCATCCAGT
	LHX3	F: ACTTGAGCCTTGTCCCTTCAG	R: TGGTCTACTTCATCCAGCCAG
	PAX7	F: AGCATCCTTAGCAACCCGAG	R: TGTAGGTGGGTGGGCAGTAA
**Human**	β-actin	F: TGGCACCCAGCACAATGAA	R: CTAAGTCATAGTCCGCCTAGAAGCA
	ACOD1	F: ATGTTGGTATTGAAGTGCAAGGC	R: TCCTAAAAACTTGGATGCAGCAG

Table S4. Antibodies used in this study.

Reagent	Cat#	Supplier	Application
IRG1	ab222411	Abcam	WB
IRG1	PA5102893	Invitrogen	IF
CD68	Ab955	Abcam	IF
F4/80	14-4801-82	Invitrogen	IF
iNOS	ab210823	Abcam	IF
iNOS	A3774	ABclonal	WB
Arg1	A4923	ABclonal	IF
CD206	A26948	Abclonal	WB
CD86	A23649	Abclonal	WB
Nrf2	A11159	ABclonal	WB
PRDM1	ab243146	Abcam	WB
JUN	A25329	ABclonal	WB
STAT5a	ab32043	Abcam	WB
STAT5b	ab178941	Abcam	WB
GFI1	31929	CST	WB/ChIP
JUND	5000	CST	WB
PAX7	ab187339	Abcam	WB
LHX3	A19590	ABclonal	WB
β-actin	HRP-66009	Proteintech	WB
